# NMR spectroscopic investigation of LiNO_3_-induced SEI modification in Li–S batteries: a concentration-dependent study

**DOI:** 10.1039/d6fd00047a

**Published:** 2026-05-18

**Authors:** Jana B. Fritzke, Mark Stockham, Marie Juramy, Samuel D. S. Fitch, Liam Furness, Nuria Garcia-Araez, Clare P. Grey

**Affiliations:** a Yusuf Hamied Department of Chemistry, University of Cambridge Lensfield Road Cambridge CB2 1EW UK Jana.beatrice.fritzke@associated.ltu.se cpg27@cam.ac.uk; b The Faraday Institution, Harwell Campus OX11 0RA Didcot UK; c School of Chemistry and Chemical Engineering, University of Southampton SO17 1BJ Southampton UK

## Abstract

The growing demand for sustainable energy has intensified efforts to develop safer, high-performance batteries. Lithium metal offers exceptional energy density but its use is limited by safety concerns and short cycle life. Electrolyte additives such as LiNO_3_ are known to enhance battery performance, yet their specific mechanism remains unclear. Nuclear magnetic resonance (NMR) spectroscopy provides a powerful and non-destructive means to probe Li metal batteries, offering a unique insight into the Li species and interfacial processes. Among the different NMR methods, *operando*^7^Li NMR measurements of the Li–S battery enable time-resolved and quantitative monitoring of electrochemical Li metal deposition, thereby linking electrochemical performance to changes in the metallic Li environment. Complementarily, *ex situ* dynamic nuclear polarization (DNP) NMR experiments on Li metal microstructures provide detailed structural information about the interface between the metal and the solid–electrolyte interface (SEI). Together, these approaches provide a comprehensive picture of both the dynamic and structural aspects governing Li metal anode behavior. In this work, we systematically investigate the influence of LiNO_3_ concentration in the ubiquitous Li–S electrolyte, 1 M LiTFSI DOL : DME, on controlling anode performance and interfacial processes using a combination of *operando*^7^Li NMR and *ex situ* DNP NMR spectroscopy. DNP NMR spectroscopy reveals that LiNO_3_ distinctly modifies the inner SEI, correlating with improved cell performance. In contrast, *operando*^7^Li NMR shows that increasing LiNO_3_ concentrations only marginally affect Li deposition. Together, these results demonstrate that while LiNO_3_ enhances Li metal anode behavior in Li–S batteries, higher additive levels do not yield additional benefits. This combined NMR approach provides new insight into interfacial processes and supports rational electrolyte design for high-performance Li–S batteries.

## Abstract 2

NMR spectroscopy reveals LiNO_3_-induced SEI restructuring, though increasing its concentration yields minimal further performance improvements.

## Graphical abstract

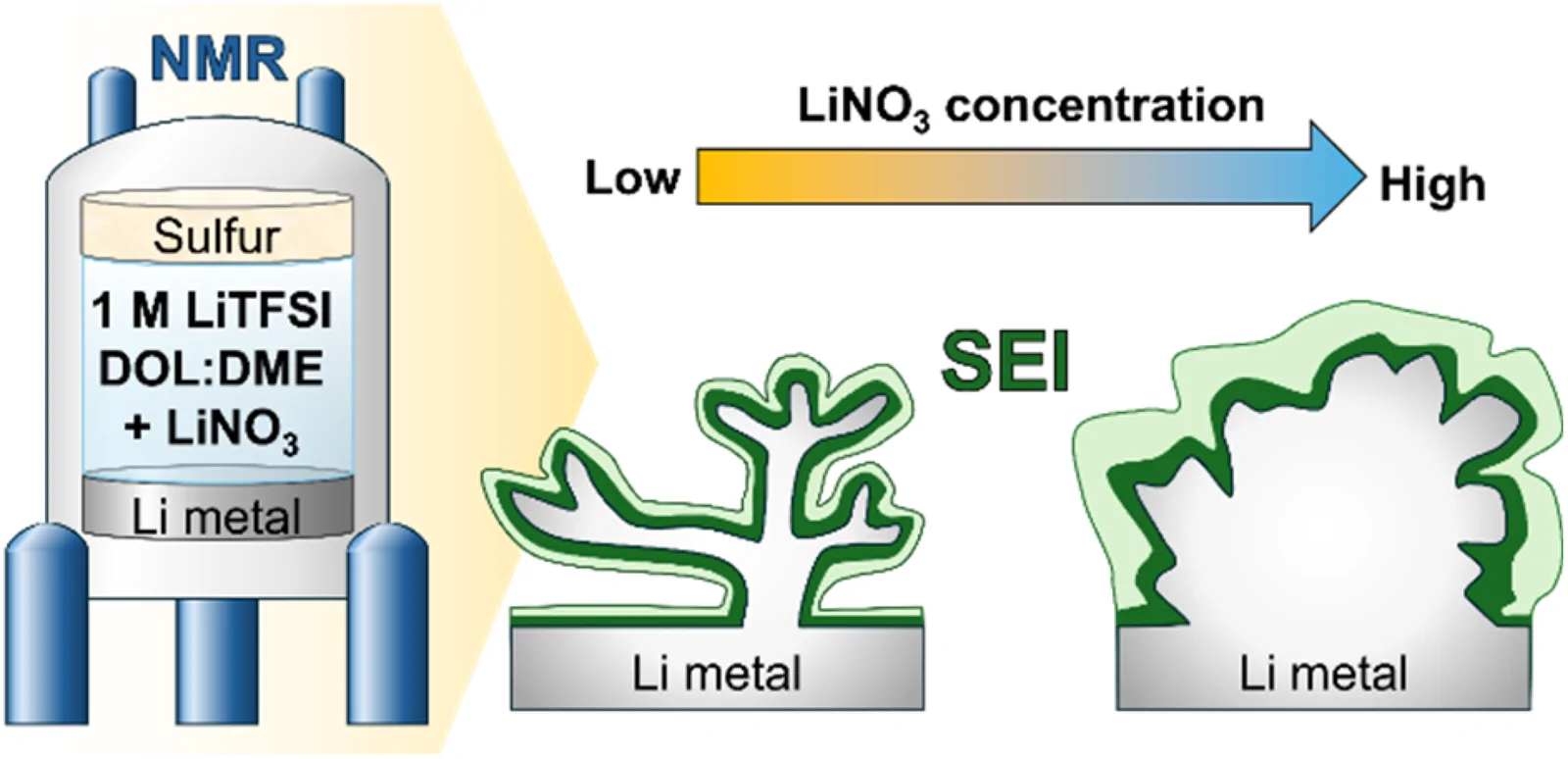

## Introduction

The substantial expansion of green energy production, intended to mitigate the excessive reliance on conventional fossil fuels, has catalyzed the advancement and widespread adoption of energy storage technologies, particularly rechargeable batteries.^1,2^ Lithium-ion batteries (LIBs) have dominated portable electronics due to their high energy density and cycling stability, in recent years. However, their capacity is constrained by the limited theoretical performance of cathode materials such as LiFePO_4_ (LFP), LiCoO_2_ (LCO) and LiNi_*x*_Mn_*y*_Co_*z*_O_2_ (NMC), making it challenging to meet growing energy storage demands.^3^ Therefore, the development of next-generation batteries with higher capacity, reduced cost, and enhanced safety is essential. Among the most promising candidates, lithium–sulfur (Li–S) batteries offer a theoretical energy density of 2600 Wh kg^−1^ and leverage the high natural abundance of sulfur, making them highly attractive for future energy storage solutions.^4,5^ Nevertheless, the practical application of Li–S batteries is severely restricted by the poor cycling performance due to the highly unstable Li metal anode.^6^ The main problems of the Li metal anode that hinder commercialization are uneven metal deposition leading to dendrite formation and the low coulombic efficiency (CE) resulting in consumption of electrolyte and active Li metal.^7^ These two problems consequently lead to short cycle life and critical safety issues, because of soft- and hard short circuit formation.^8^

Many strategies for preventing dendrite formation concentrate on enhancing the stability and uniformity of the solid electrolyte interphase (SEI) on the Li metal surface, typically by tailoring electrolyte composition and incorporating additives that promote controlled SEI development.^9^ An ideal SEI should facilitate efficient Li^+^ transport while maintaining electronic insulation to prevent electron-induced electrolyte reduction. Therefore, a uniform morphology and composition of the SEI is required to ensure a homogeneous current distribution. Furthermore, this layer should be elastic and flexible so as to maintain its integrity during the volume changes that occur upon plating and stripping of the Li metal due to the formation of microstructured Li metal.^10^

In the context of improving the cycling performance, LiNO_3_ has attracted significant attention as a key additive in electrolytes for Li metal batteries, especially in Li–S systems, since its introduction in 2008 by Mikhaylik.^11^ However, the working mechanism of LiNO_3_ inside of the battery remains a topic of intense debate.^12,13^ One popular explanation is that LiNO_3_ forms a protective SEI on the surface of the Li metal anode and prevents irreversible interaction of polysulfides (poly-S) with the Li metal, the so-called poly-S shuttle.^14^ In particular, recent reports indicate that the SEI formed in LiTFSI/LiNO_3_ salt mixtures in ether-based solvents, such as 1,3-dioxolane (DOL) and 1,2-dimethoxyethane (DME), is thicker than the SEI on Li metal compared to LiTFSI alone.^15^ This can be explained by the SEI-forming capabilities of LiNO_3_, as it can spontaneously react with Li metal and decompose into the reduction products, Li_2_O, Li_3_N and LiN_*x*_O_*y*_.^16,17^ While LiNO_3_ and related electrolyte formulations have shown promise in modifying SEI chemistry and improving interfacial stability, the resulting SEI is often complex and heterogeneous, influenced by multiple decomposition pathways of salts and solvents. Such variability can lead to local inhomogeneities that critically affect Li deposition behavior.^18^ Understanding these morphological and chemical changes requires advanced characterization techniques to probe SEI structure and Li metal evolution. To examine Li metal morphology and performance during plating various characterization methods have been used, including scanning electron microscopy (SEM),^19,20^*operando* optical microscopy^2^ and transmission electron microscopy (TEM).^21^ Furthermore, the surface chemical composition of the SEI on Li metal has been analyzed using X-ray photoelectron spectroscopy (XPS),^22^ its top layer morphology characterized by cryogenic electron microscopy^23,24^ and its thickness measured *via* atomic force microscopy (AFM).^15,25^ Although these advanced techniques have significantly improved our understanding of Li metal plating and the corresponding SEI composition and structure, establishing definitive correlations between structure, properties, and electrochemical performance remains a major challenge.

NMR spectroscopy is a powerful tool to detect the local environment around nuclei such as ^7^Li and connect these, *via* quantitative measurements, with the various dynamic electrochemical processes by performing *operando* investigations of metal plating and corrosion.^26–28^ These *operando* studies in combination with detailed *ex situ* analysis of the interface with high chemical and, in certain scenarios, spatial resolution can provide in depth understandings of the performance–structure relationships.^29^ Especially, magic angle spinning dynamic nuclear polarization (MAS DNP) NMR spectroscopy, a method using the polarization transfer from electron spins of Li metal to the surrounding nuclear spins *via* the Overhauser effect, enables selective detection of SEI components close to the electrode surface.^30^

Although it is well established that LiNO_3_ is critical in preventing the poly-S shuttle, to the best of our knowledge, a careful study on the effect of this salt's concentration on the performance of Li metal plating and full Li–S batteries considering SEI properties has yet to be reported. Previous reports have indicated a concentration-dependent effect of LiNO_3_ on Li–S cycling behavior and have highlighted the challenges in correlating performance with SEI chemistry.^31,32^ To address this research gap, we conducted the first systematic study of the effect of LiNO_3_ concentration in 1 M LiTFSI DOL : DME electrolyte combining *operando* and DNP NMR spectroscopy with electrochemical methods to elucidate how additive levels influence anode performance and interfacial processes. This allows a detailed spectroscopic characterization of how interfacial changes manifest at the cell level under practical cycling conditions. Based on a survey of the Li–S literature, we selected electrolytes containing 1 M LiTFSI with 0.25 M, 0.8 M and 1 M LiNO_3_, as well as 0.375 M LiTFSI + 0.625 M LiNO_3_ and an additive-free reference system.^33–37^*Operando*^7^Li NMR measurements during Li metal plating indicate that increased LiNO_3_ concentration has only a limited influence on microstructure formation. While, DNP NMR shows a clear modification of the inner SEI upon the introduction of LiNO_3_ in the electrolyte, consistent with the observed enhancement in full-cell performance. The combination of these results provides a thorough understanding of the relationship between additive concentration, interface structure and cell behavior and, thereby, offering a new basis for rational electrolyte design for high-performance Li–S batteries.

## Methods

### Li–Li cells

Cell assembly and air-sensitive material handling were done in an argon glovebox (MBraun, O_2_, H_2_O < 1 ppm). The electrolytes were prepared using 1 M lithium bis(trifluoromethanesulfonyl)imide (LiTFSI, Sigma-Aldrich, 99.95%) in 1,3-dioxolane (Acros Organics, anhydrous, 99.8%) and 1,2-dimethoxyethane (Merck, anhydrous, 99.9%) (DOL : DME in 1 : 1 volume ratio) and different concentrations of lithium nitrate (LiNO_3_, Alfa Aesar, 99.999%). The LiTFSI and LiNO_3_ salt were dried for 20 h at 120 °C under vacuum before use.

For the preparation of the polysulfide containing electrolyte, solid sulfur (S_8_, 100 mesh, sublimed, Sigma-Aldrich, dried under vacuum at room temperature for three days) and lithium sulfide (Li_2_S, 99.98%, Sigma-Aldrich, dried under vacuum at 40 °C for three days) were added to the electrolyte in an appropriate molar ratio to produce Li_2_S_6_ as follows:^38^5/8 S_8_ + Li_2_S → Li_2_S_6_

The mixture was heated to 60 °C with stirring for a week, leading to complete dissolution. The poly-S concentration is 1 mole of atomic sulfur, in the form of poly-S species, in one liter of solution.

Lithium metal disks (15.6 mm × 0.25 mm thick) were purchased from PI-KEM, opened, stored in an argon glovebox, and used as received. Stainless steel 2032 coin cell parts (Cambridge Energy Solutions) were sonicated in ethanol and dried at 60 °C overnight prior to cell assembly. Glass fibre (Whatman GF/B or Advantec, GC-50) separators were used after being dried in vacuum at 100 °C overnight and Celgard 3501 separator was dried under vacuum at 40 °C for 20 h.

Coin cells consisting of a two-part case (2032), a conical spring, two 0.49 mm stainless steel disks, two Li electrodes, and a glass fibre separator (Whatman GF/B) with 100 µL electrolyte were used for electrochemical characterization. A BioLogic VSP-300 cycler was used for impedance spectroscopy. EC-Lab software (V11.32) was used for data collection and processing. To detect the native SEI evolution potentiostatic electrochemical impedance spectroscopy (PEIS) measurements were conducted every 1 h using a frequency range between 1 MHz and 0.1 Hz at 10 mV amplitude.

### Li–Ni cells

All electrolyte preparation and electrochemical cell assembly were done inside an argon-filled glovebox (Unilab, MBraun, H_2_O and O_2_ < 0.1 ppm). CR2032 coin cells were used (Seika Sangyo GmbH Germany). These were sealed using a HS-HCR2 hand-operated coin crimper (Seika Sangyo GmbH Germany).

For electrolyte preparation, LiTFSI (lithium bis(trifluoromethanesulfonyl)imide, Sigma-Aldrich, anhydrous, 99.99%) and LiNO_3_ (lithium nitrate, Sigma-Aldrich, 99.99%) were dried at 120 °C under vacuum overnight. DME (1,2-dimethoxyethane, Sigma-Aldrich, anhydrous, 99.5%) and DOL (1,3-dioxolane, Sigma-Aldrich, anhydrous, 99.8%) had safesure caps, which were removed in the glovebox. DME and DOL were further dried with 4 Å molecular sieves (8–12 mesh bead size, Aldrich). The sieves were first activated *via* heating under vacuum in a Büchi tube at 220 °C for 72 h. The tube was then sealed and transferred to a glovebox, where the sealed tube was left to cool overnight before the sieves were used. The salt concentrations in the electrolytes are reported as moles of salt per litre of solvent mixture (Table S1).

For cell preparation, a 15 mm-diameter lithium disc (battery grade, China Energy Lithium Co., 100 microns thick) was pressed into a 0.5 mm coin cell spacer and placed inside the gasket side of the coin cell case. Two 16 mm glass fiber grade F separators (pre-dried in a Büchi oven at 140 °C for at least two days, afterwards transferred into the glovebox under vacuum) were placed on top of the metal disc and wetted with 120 µL of electrolyte. A Ni disc (15 mm) was then placed on top with a 0.5 mm spacer and a wave spring. The coin cell cap was added, and the cell was crimped closed.

Cells were tested on either a BioLogic VMP3 or VMP2 unit at 25 °C in either a Memmert or Binder climate chamber. All cells were initially rested for 6 h at the open circuit voltage (OCV), followed by three initial pre-conditioning Li plating and stripping steps at 0.25 mA cm^−2^ (0.38 mA) for 15 min each, with 5 min rest in between. The plating and stripping behavior was subsequently investigated at 1 mA cm^−2^ (1.54 mA) for 10 h, with a 20 min rest between steps.^39^

### Li–S batteries

Sulfur electrodes were fabricated following a previously reported procedure.^40^ The electrode composition (by weight) consisted of 65% sulfur (Sigma-Aldrich, 99.98%), 21% Ketjenblack EC-600JD (NanoGrafi), 3.5% Super C65 (Imerys), 3.5% carbon nanofibers (20–200 nm × 100 µm, Sigma-Aldrich), 5.6% poly(ethylene oxide) (PEO, *M*_w_ ≈ 4 000 000, Sigma-Aldrich), and 1.4% polyvinylpyrrolidone (PVP, *M*_w_ ≈ 360 000, Sigma-Aldrich).

To prepare the sulfur–carbon composite, sulfur and the carbon materials were first manually blended using a mortar and pestle. The mixture was then heated in a lidded porcelain crucible (wrapped in foil) at 155 °C for 1–2 h to melt-infiltrate the sulfur into the carbon matrix. After cooling, the composite was ground again for approximately 5 min.

The composite was subsequently mixed with the PEO and PVP binders, and a viscous slurry was prepared using ultrapure water (18.2 MΩ) containing 7.5 wt% ethanol (Fisher Scientific, 99.7%). Approximately 3.3 mL of solvent per gram of solids was used. The slurry was homogenized in a Thinky mixer (ARE-250) at 1300 rpm for 5 min, followed by 1800 rpm for 5 min and 2000 rpm for 5 min, with 5 min cooling intervals between each mixing step.

The resulting slurry was cast onto carbon-coated aluminium foil (MTI) using a doctor blade. Circular electrodes (14 mm diameter) were punched out and dried under vacuum at room temperature in a Büchi tube for at least two days.

Coin cell assembly followed the previously described procedure, substituting sulfur electrodes for Ni discs and using Celgard 2400 as separator. The Celgard separators were cut into 16 mm discs and dried under vacuum at 60 °C for 72 h before use.

The electrochemical testing protocol was as follows:^41^ cells were allowed to equilibrate at OCV for 6 h and then a precondition cycle, in which the Li–S cells were discharged at C/50 (*C* = 1672 mAh g_S_^−1^) to 1.9 V and charged at C/25 to 2.8 V. Then, the electrochemical performance of the Li–S cells was tested at C/10 between the voltage limits of 1.8 V and 2.6 V, respectively, at C/10. All the measurements were performed at 25 °C inside a climatic chamber (Memmert).

### SEM investigation

After galvanostatic plating for 10 h with a current density of 0.5 mA cm^−2^, the *operando* capsule cells were transferred into an Ar glovebox and disassembled. The plated Li metal electrode was mounted onto the SEM stage of the transfer module (Kammrath & Weiss, type CT0). The electrodes were not rinsed with a solvent before the measurement. The samples were transferred into the SEM chamber using the air sensitive transfer module under an inert atmosphere (Ar), without being exposed to air. SEM images were acquired with a Tescan MIRA3 FEG-SEM instrument at an acceleration voltage of 5.0 kV.

For the SEM characterization of the coin cells, the cells were disassembled in the glovebox and the electrodes were rinsed with ∼3 mL of DME to remove salt and poly-S residues. The solvent was then allowed to dry off in the glovebox and the electrodes were stored face up in glass vials. The cycled electrodes were loaded into a SemiLab sample transfer case and reopened under vacuum inside the transfer chamber of the SEM (Zeiss Sigma 500 VP FESEM).

### Measurement of conductivity and viscosity

The conductivity of the electrolyte solutions was measured *via* impedance measurements in a conductivity cell (High Temperature Conductivity Cell, HTCC, BioLogic) inside a climatic chamber (Memmert) at 25 °C. The conductivity cell was calibrated using a 0.1 M KCl standard solution.

The viscosity of the electrolyte solutions was evaluated using a Cannon-Fenske viscometer. The kinematic viscosity is given by the product of the efflux time and the viscometer constant (in this case, 0.003253 ± 0.000011 mm^2^ s^−2^). The dynamic viscosity equals the product of the kinematic viscosity and the density of the solution. The density of the solutions was determined from the mass of the solution contained in a 5 mL volumetric flask. Table 1 and Fig. S6 summarize the results of the evaluation of these electrolyte properties.

**Table 1:** Values of kinematic and dynamic viscosity and conductivity of the electrolyte compositions studied in this work

Electrolyte	Kinematic viscosity/cSt	Dynamic viscosity/mPa	Conductivity/mS cm^−1^
1 M LiTFSI	1.15 ± 0.03	1.27 ± 0.04	12.25 ± 0.12
1 M LiTFSI + 0.25 M LiNO_3_	1.29 ± 0.03	1.43 ± 0.05	11.74 ± 0.01
1 M LiTFSI + 0.8 M LiNO_3_	1.62 ± 0.04	1.81 ± 0.07	10.31 ± 0.10
1 M LiTFSI + 1 M LiNO_3_	1.81 ± 0.03	2.04 ± 0.06	9.94 ± 0.10
0.375 M LiTFSI + 0.625 M LiNO_3_	0.97 ± 0.02	1.00 ± 0.03	6.56 ± 0.07

### *Operando* NMR spectroscopy

The *operando* NMR experiments were conducted on a Bruker Avance 300 MHz spectrometer (the Larmor frequency for ^7^Li being 116.6 MHz) using a solenoidal Ag-coated Cu coil. The spectra were recorded using an *in situ* automatic-tuning-and-matching probe (ATM VT X *in situ* WB NMR probe, NMR Service, eProbe) that allows for an automatic recalibration of the NMR rf-circuit during an *in situ* electrochemistry experiment. The retuning of the rf-circuit becomes essential in order to quantify the NMR signals when the sample conditions are changing during the electrochemistry.^42^ The probe has highly shielded wire connections to the electrochemistry with low-pass filters (5 MHz) attached to the probe, minimizing the interferences between the NMR- and the electrochemistry circuit. Overall, the *in situ* setup allows for highly reproducible NMR measurements.

Single-pulse experiments were used to collect the NMR data, with a recycle delay of 1 s (>5 × *T*_1_) and 128 transients recorded. This resulted in an experimental time of about 2.5 min. The shift of ^7^Li was referenced to 1 M LiCl in water at 0 ppm. The spectra were processed in the Bruker Topspin software using the automatic phase and baseline correction. Further data processing was done in R. The total intensity of the Li metal peak was integrated over the ^7^Li shift range of 310–220 ppm and normalized to the intensity measured at the beginning of the experiment. Due to the use of metallic electrodes in the cell, the skin depth effect must be considered when NMR spectra of lithium cells are recorded. To detect any change in the surface area, the total metal peak (220 to 310 ppm) was integrated and plotted as “total metal” in this paper. The formed microstructure during the deposition, however, is reported in the literature to be smaller than the skin depth, and therefore, the intensity of the microstructure peak is directly proportional to their mass. The quantification of the metal signals followed the theory developed and described by Bhattacharyya *et al.*^43^

For the *in situ* NMR cell setup in this study, all the cells operate with galvanostatic plating at the applied current densities of 1 mA cm^−2^ and 0.5 mA cm^−2^ using a BioLogic VSP cycler. The calculated limiting current density is ∼7 mA cm^−2^.^2^ Thus, the galvanostatic experiments performed in this work are at the low current density regime.^44^ A capsule cell (NMR Service, eProbe) made out of PEEK (polyether ether ketone) was used for all *in situ* NMR experiments and has been described before.^28^ Symmetrical cells consisted of two Li metal electrodes (0.5 × 1 cm) on Cu mesh current collector. Two sheets of glass fiber (Advantec GC-50) were soaked with 150 µL electrolyte.

*Operando* corrosion experiments were measured using a bare Cu foil anode (acetic acid-treated^26^) and a lithium iron phosphate cathode (LFP, PI-KEM, ∼2.2 mAh cm^−2^ areal capacity). The electrolyte (100 µL) was soaked into the glass fiber (Whatman GF/B) and Celgard separators. Li metal plating on Cu was done at 1 mA cm^−2^ for 2 h and afterwards the cell was rested at OCV.

### SEI characterization with dynamic nuclear polarization (DNP) magic angle spinning (MAS) NMR spectroscopy

Individual samples were prepared by galvanostatic plating in Li//Li symmetric coin cells (2032, Cambridge Energy Solutions) with Celgard 3501 and glass fiber (Whatman GF/B) separators at 0.5 mA cm^−2^ for 100 h. After plating, the electrode was dried under vacuum for 0.5 h and afterwards the microstructural Li was removed from the electrode surface using a spatula. Microstructural Li was mixed thoroughly with dried KBr (40 h at 200 °C) (10 : 1 w/w) using a mortar and pestle in the glovebox to limit electrical connectivity of conductive Li particles and reduce eddy currents^45^ while spinning the sample in the NMR magnet before packing into a 1.3 mm (ZrO_2_) or 3.2 mm (sapphire) rotor.

All DNP MAS NMR experiments were performed on a Bruker Avance NEO 400 MHz (9.4 T) spectrometer equipped with a 3.2 mm HFXY DNP MAS Bruker and a 1.3 mm HXY DNP MAS Bruker probe head. All spectra were collected at room temperature. The rotor spinning was set to 10 or 12.5 kHz for 3.2 mm and 30 kHz for 1.3 mm rotors. A klystron microwave source operating at 264 GHz with a power set to 5.2 W was used in all experiments. All spectra were collected with microwaves on and off to calculate the enhancement factor from the integrals (*ε* = *A*_on_/*A*_off_).

^7^Li Hahn-echo experiments were measured with a recycle delay of 5 s, and 128 scans. ^7^Li–^19^F cross polarization magic-angle spinning (CPMAS) measurements were performed with a ramped (50–100%) pulse on ^7^Li, a contact time of 0.6 ms, a recycle delay of 5 s, and 1024 scans. ^7^Li–^1^H CPMAS experiments were performed with a contact time of 1.5 ms, a recycle delay of 5 s, and 256 scans. ^1^H NMR was externally referenced to adamantane at 1.8 ppm, ^7^Li and ^19^F were externally referenced to LiF at −1 ppm and −204 ppm, respectively.

## Results

### Electrochemical characterization of Li metal plating

**Fig. 1:**
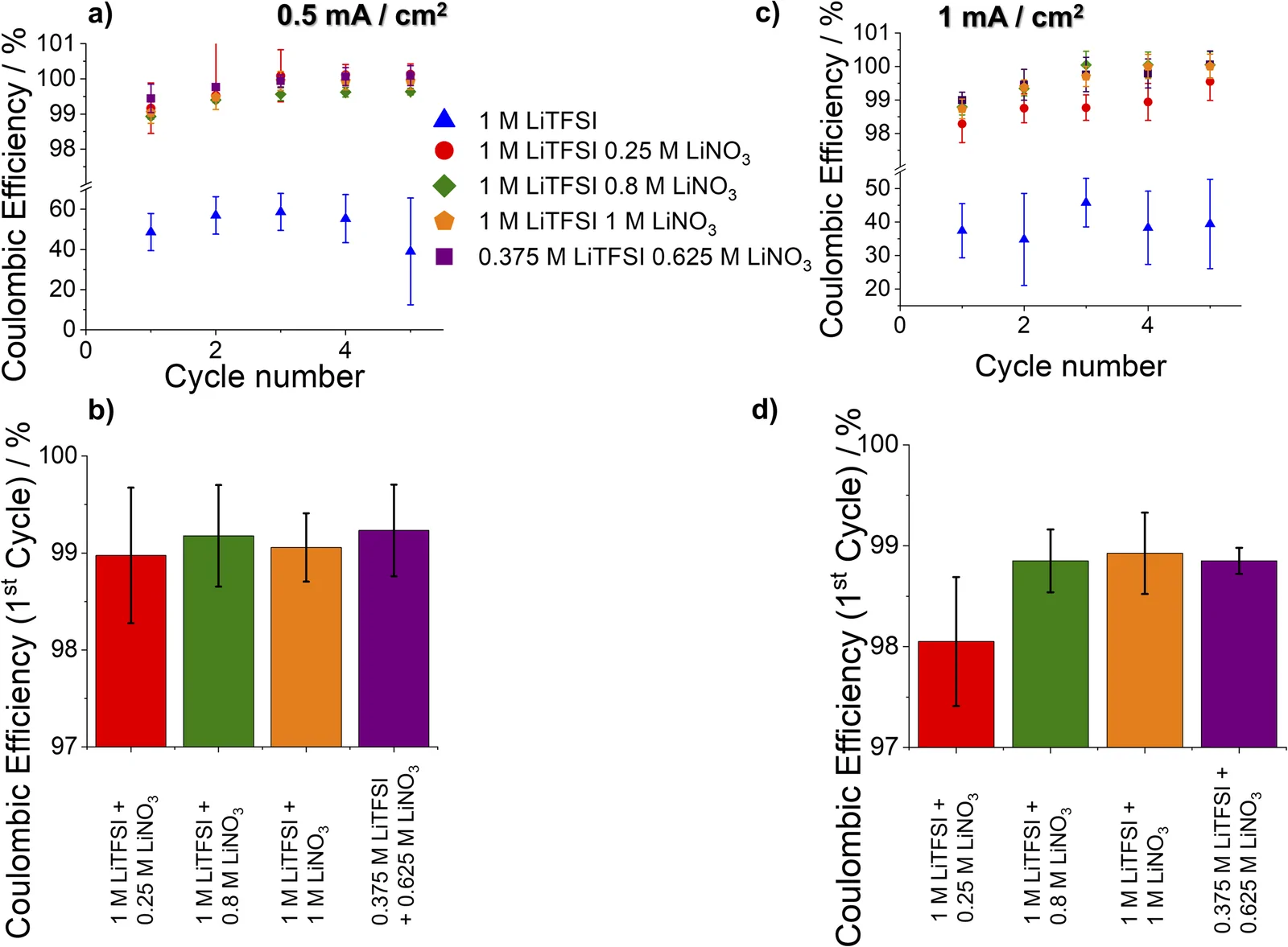
Coulombic efficiencies of Li–Ni cells during first 5 cycles at (a) 0.5 mA cm^−2^ and at (c) 1 mA cm^−2^ with the different electrolytes: 1 M LiTFSI (blue), 1 M LiTFSI + 0.25 M LiNO_3_ (red), 1 M LiTFSI + 0.8 M LiNO_3_ (green), 1 M LiTFSI + 1 M LiNO_3_ (orange) and 0.375 M LiTFSI + 0.625 M LiNO_3_ (purple) in DOL : DME. Comparison of the coulombic efficiency of the first cycle at (b) 0.5 mA cm^−2^ and (d) 1 mA cm^−2^ in the different LiNO_3_-containing electrolytes.

Li anode performance in the selected electrolytes was investigated *via* Li metal plating and stripping on a Ni inert support, using a Li metal anode counter electrode. The experiments were performed at two different current densities: 0.5 mA cm^−2^ and 1 mA cm^−2^ (Fig. 1, repeats in Fig. S4 and S5), which correspond to C-rates of C/5 and C/10 for Li–S cells with sulfur loading of 3 mg cm^−2^. Previous work has shown that this sulfur loading is sufficient to produce high energy Li–S batteries,^46^ and the moderate C-rates used here are relevant for the most promising Li–S battery applications.^47^

Fig. 1 shows that the coulombic efficiency of the Li plating/stripping experiments is very low in the electrolyte without LiNO_3_, evidencing that significant side-reactions accompany the lithium plating process, and consequently, the stripping capacity is a small fraction (less than 50%) of the plating capacity (Fig. 1a and b). Such severe mismatch between plating and stripping capacities can be attributed to issues of exacerbated SEI formation, resulting from lithium plating morphologies with high surface area, as well as the formation of dead lithium, which is also promoted by dendrite formation. As will be shown later, the formation of lithium microstructures is intrinsically caused by inhomogeneities in the lithium SEI compositions.

All electrolyte formulations containing LiNO_3_ show high coulombic efficiency, with minor variations between different electrolyte compositions. This shows that, as long as LiNO_3_ is present in the cell, the Li metal anode electrochemistry benefits from a high coulombic efficiency, regardless of the LiNO_3_ concentration. This is presumably due to the promotion of flatter lithium deposits in the presence of LiNO_3_, which is promoted by the more beneficial SEI properties, as will be discussed below (Fig. S8). A close inspection of Fig. 1 shows a small increase in the coulombic efficiency when the specific current is decreased from 1 mA cm^−2^ to 0.5 mA cm^−2^, as expected for the formation of flatter deposits as the current density decreases (Fig. 1c and d). However, the variation in coulombic efficiency values is small, indicating that cells with LiNO_3_ can cycle well for all electrolytes containing LiNO_3_.

### *Operando* NMR spectroscopy

**Fig. 2:**
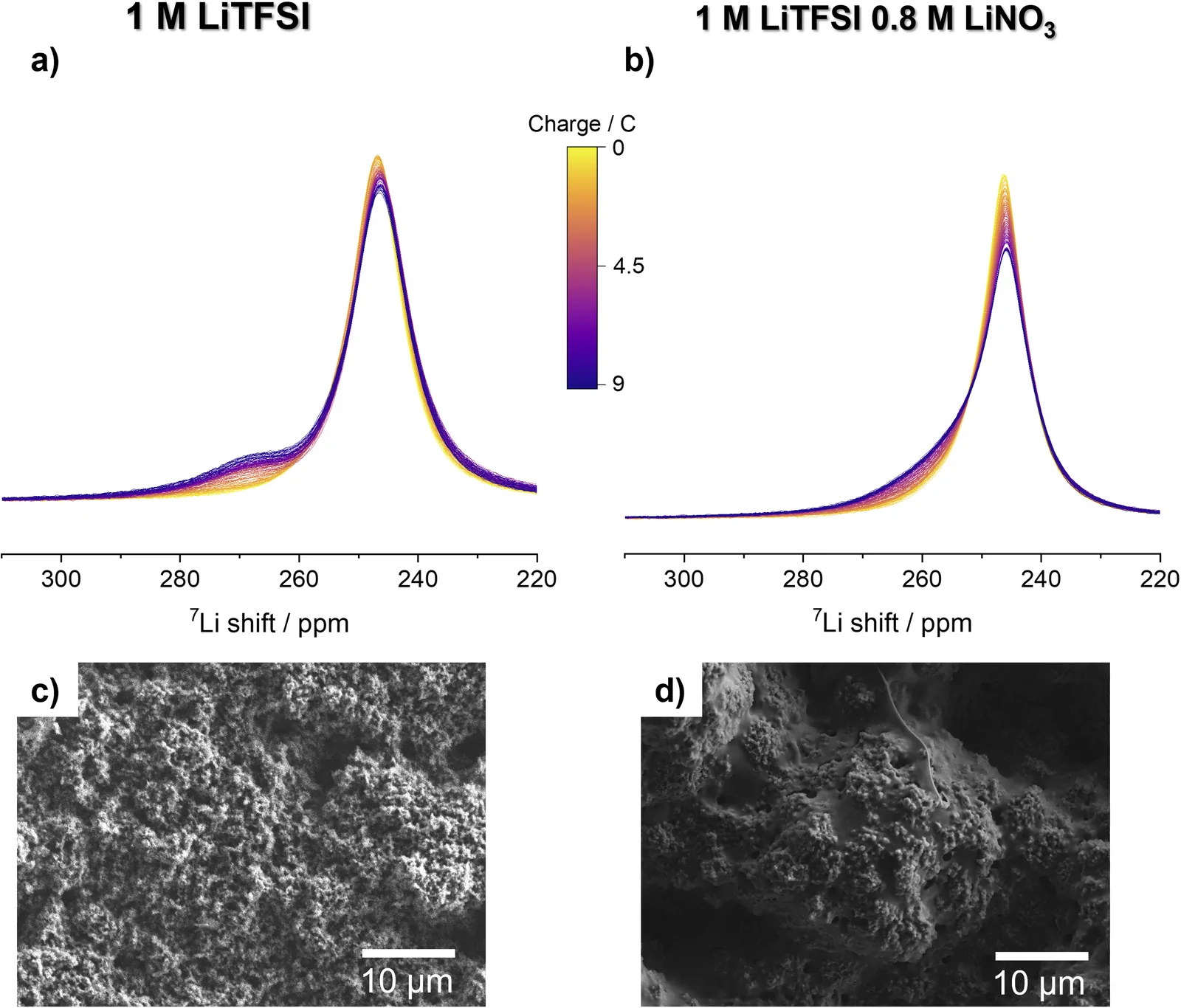
*Operando* NMR experiments of galvanostatic plating at 0.5 mA cm^−2^ in symmetrical Li metal cells. On the top, change of intensity and shape of the ^7^Li NMR metal signal, and on the bottom, SEM images of the Li electrodes extracted from the same *operando* NMR cells at the end of the NMR experiment for (a and c) 1 M LiTFSI and (b and d) 1 M LiTFSI + 0.8 M LiNO_3_ in DOL : DME.

*Operando* ^7^Li NMR measurements of symmetrical Li metal cells were performed to investigate the morphological changes induced in the Li metal electrode during unidirectional galvanostatic plating. Fig. 2a and b show the *operando*^7^Li NMR spectra in the region where the signal of metallic lithium appears. Significant changes in intensity and lineshape occur during the experiment. These changes can be explained by three effects: changes in surface area, the skin depth and the bulk magnetic susceptibility (BMS) effects. The applied radiofrequency (rf) during the NMR measurement penetrates the Li metal electrodes to a certain depth (*ca.* 12 µm for Li), which is called the skin depth, and this influences the detected signal intensity.^48^ Hence, the detected Li metal integral is sensitive to the surface area of the electrodes. The SEM images of the electrodes, obtained after the *operando* NMR measurement, show that the Li metal microstructures are smaller than the skin depth (Fig. 2c and d), therefore, the increase in the integral of the Li metal peak can be directly correlated to the amount of plated Li metal. The BMS effect can be used to differentiate the bulk, *i.e.*, dense Li metal, and the various plated microstructures such as dendritic and mossy morphologies, as the local fields of Li metal depend on the shape, packing and orientation with respect to the static magnetic field.^43^ In the orientation of the electrode within the cell used in this experiment, the plated Li microstructures show a higher shift than that of the bulk metal resulting in a new peak (at ∼260–275 ppm) or a broadening of the metallic peak while plating (Fig. 2a, b and S7). Hence, the BMS effects allow a decomposition of the metal peak and accordingly a quantification of the microstructures, as previously reported by Bhattacharyya *et al.*^43^

Closer inspection of Fig. 2a reveals that the microstructures formed without LiNO_3_ show a shift of 269 ppm, and the SEM image (Fig. 2c) reveals thin, mossy morphologies of the plated Li. In comparison, NMR spectra with electrolytes containing LiNO_3_ show broadening of the Li metal peak resulting from a microstructure peak with a shift of 258 ppm (Fig. 2b) and the corresponding SEM (Fig. 2d) shows a dense morphology of the plated Li metal. These variations in the NMR signal are fully consistent with the findings by Chang *et al.* who showed that Li morphologies growing away from the electrode surface are characterized by larger chemical shifts (∼270 ppm) than microstructures close to the surface (∼260 ppm).^49^

**Fig. 3:**
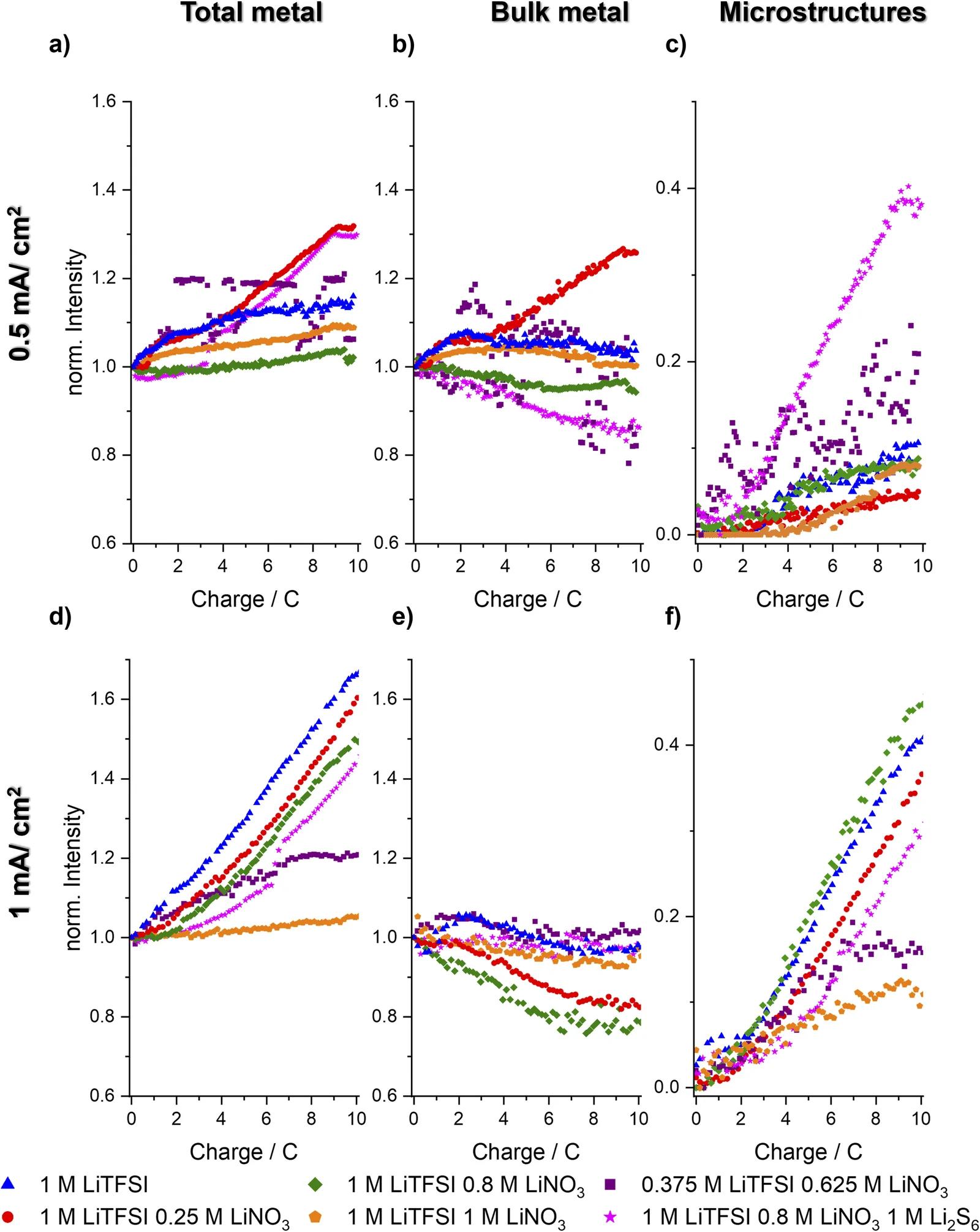
Evolution of integrals from *operando*^7^Li NMR spectroscopic measurements of unidirectional Li-metal plating in symmetrical cells in different electrolytes. Normalized integrals of the full metal peak between 220 and 310 ppm denoted as “Total metal” (left column, a and d), and the normalized integrals of the deconvoluted bulk (middle column, b and e) and microstructured (right column, c and f) metal peak, are plotted against the applied charge during galvanostatic plating at 0.5 mA cm^−2^ (top row, a–c) and 1 mA cm^−2^ (bottom row, d–f).

Fig. 3 summarizes the results of the quantitative analysis of *operando*^7^Li NMR measurements of unidirectional Li plating in electrolytes with different amounts of LiNO_3_, and one electrolyte containing added poly-S (1 M Li_2_S_6_). Significant variations in the evolution of the intensity of the total metallic Li signal (normalized to the initial integral) integrated from 220–310 ppm and the decomposed integrated signals for the bulk (at ∼250 ppm) and microstructural (at ∼260–275 ppm) Li metal were observed. In all experiments, the total Li metal signal increases with time, which can be correlated with the formation of higher Li metal electrode surface areas by the deposition of microstructural Li metal and pitting of the stripped electrode (Fig. 3a and d). This can be correlated with the increase of the microstructure peak in all experiments; however, the electrolyte and the applied current density significantly influence the amount of detected microstructures. The bulk metal signal shows a decrease in integral which is ascribed to a skin depth effect (Fig. 3b and e). This means, the formation of a dense microstructural Li metal coating on the surface shields the rf so that it can no longer penetrate the bulk to the same depth as at the beginning of the experiment. At 0.5 mA cm^−2^ the highest amount of microstructures can be detected in electrolytes with the following compositions: 1 M LiTFSI + 0.8 M LiNO_3_ + 1 M Li_2_S_6_ and 0.375 M LiTFSI + 0.625 M LiNO_3_ (Fig. 3c).

The enhanced growth of Li microstructures in the poly-S containing electrolyte (Fig. S7) can be attributed to alteration in the SEI composition. It was previously reported that poly-S contribute to the SEI formation forming Li_2_S, which is a poor ionic conductor.^50^ This can generate inhomogeneities in the ionic conduction on the surface of the electrode leading to nucleation hot-spots and dendritic growth. The microstructure formation in the electrolyte with 0.375 M LiTFSI + 0.625 M LiNO_3_ can, at least in part, be attributed to the lower ionic conductivity (see Fig. S6) of this electrolyte, and indeed, previous work concluded that enhancing the electrolyte conductivity is critical to prevent concentration gradients in the cell and dendritic growth.^51^

The other electrolyte compositions show very similar microstructure formation (Fig. 3c). The electrolyte without LiNO_3_ shows deposition of both bulky metal and microstructures until a total charge of 3C. After that, microstructural plating dominates. In the electrolyte with 0.25 M LiNO_3_, bulky and microstructural metal plating is detectable throughout the entire experiment and the highest concentrations of LiNO_3_ (0.8 M and 1 M) show until 4C only bulky deposition and after that microstructure formation. These results evidence that the addition of LiNO_3_ has an only minor effect on the microstructure formation at low current densities.

Electrochemical Li metal deposition with higher current density of 1 mA cm^−2^ shows a different behavior (Fig. 3d–f). All electrolyte compositions form microstructures from the beginning of the experiment indicated by the decrease in bulky metal and an increase in the microstructural peak. The amount of plated microstructures is similar in all electrolytes until 3C. With a higher charge passed the amount of microstructure formed changed depending on the electrolyte. The formulations without LiNO_3_, those with 0.25 M, 0.8 M LiNO_3_ and the poly-S containing electrolytes reveal a constant formation as indicated by a linear increase of microstructure peak reaching between 20 and 40% until the end of the experiment.

The poly-S containing electrolyte exhibits comparable performance at 1 mA cm^−2^ to that at 0.5 mA cm^−2^ (Fig. 3d–f). This behavior likely arises from the ability of poly-S species to corrode dendritic or mossy Li deposits, which can promote smoother Li deposition when the poly-S concentration and applied current density are properly balanced. The 1 M LiNO_3_ electrolyte showed the lowest amount of microstructures throughout the experiment, with bulky plating at the end of the experiment, revealing that at higher current densities a high concentration of the additive is beneficial for smooth Li metal plating. In the electrolyte with 0.375 M LiTFSI + 0.625 M LiNO_3_, however, no further microstructure formation is detectable after 6C have been passed. As the total metal and bulky metal peak also stay constant and no further metal plating occurs, a (soft-) short circuit has likely formed, indicating a severe cell degradation.^52^ Comparing the experiments at different current densities reveals that with higher current density more microstructures are generated with the same amount of charge passed independently from the LiNO_3_ concentration (Fig. 3c and f).

The deposition rate increases with applied current according to Faraday's law of electrolysis, but the overpotential is strongly dependent on the rate at which the Li^+^ ions reach the electrode. At higher current the supply of Li^+^ ions to the surface is limited by the rate of ion transport, whose magnitude also affects the bulk electrolyte properties of conductivity and viscosity (Fig. S6). If ion transport is hindered, a Li^+^ ion depletion near the surface causes concentration polarization promoting microstructure formation and dendritic growth. The presence of poly-S increases electrolyte viscosity and decreases the conductivity,^53^ which explains the higher amount of microstructure formed at 0.5 mA cm^−2^. In addition, the electrolyte containing 0.375 M LiTFSI + 0.625 M LiNO_3_ exhibits lower conductivity than all other electrolytes, providing a plausible explanation for cell failure at 1 mA cm^−2^.

An inhomogeneous SEI causes uneven current distribution creating localized regions of higher current density favoring dendritic plating. This non-uniform deposition increases the surface area and can rupture the SEI, expose fresh lithium and trigger further SEI formation, which causes electrolyte consumption. Consequently, SEI conductivity, mechanical flexibility and healing behavior – properties governed by the chemical composition – play a critical role in determining plating performance and needs further investigation.

### SEI characterization by DNP MAS NMR spectroscopy

The amount and morphology of the plated Li microstructures is ultimately determined by the SEI composition and structure. It is assumed that dendritic structures are related to non-uniform Li^+^-ion transport properties of the SEI creating plating hot-spots.^54^ Elucidating the impact of electrolyte additives on SEI formation and, consequently, on Li microstructure plating, requires detecting the chemical species and their spatial organization within the SEI. However, the SEI's inherent complexity and limited quantity pose significant analytical challenges. Among the few techniques capable of addressing these challenges, Overhauser (OE) DNP NMR is a unique and powerful approach.^55^ Developed to overcome the intrinsic low sensitivity of conventional NMR, DNP can enhance signal intensity by several orders of magnitude.^56^ When applied to Li metal, OE DNP leverages polarization transfer from conduction electrons in the metal to surrounding nuclei, including both nuclei in the Li metal and those within the SEI.^30,57^ For this transfer to occur, the sample must be irradiated with microwaves at a specific frequency to fulfil the matching conditions required for polarization transfer.^56^ It is common practice to record NMR DNP measurements both with and without microwave irradiation (MW). This approach allows identification of which signals are enhanced by the polarization transfer and quantifies the efficiency of the process by comparing the spectra recorded under both conditions. The ratio between the integrated signals with and without microwave irradiation is referred to as the DNP enhancement (*ε*) and is conventionally used to estimate the efficiency of the DNP process. The efficiency of the OE DNP enhancement is dependent on the distance of the species from the conduction electrons:^57^ a relatively high enhancement is representative of species closer to the Li metal, while a low enhancement indicates that the species is farther away within the SEI layer. No enhancement suggests that the species is completely outside the polarization transfer range and thus even farther from Li metal. This capability enables OE DNP to probe the inner SEI layer on Li metal dendrites, providing valuable insights into its chemical composition and spatial organization.

**Fig. 4:**
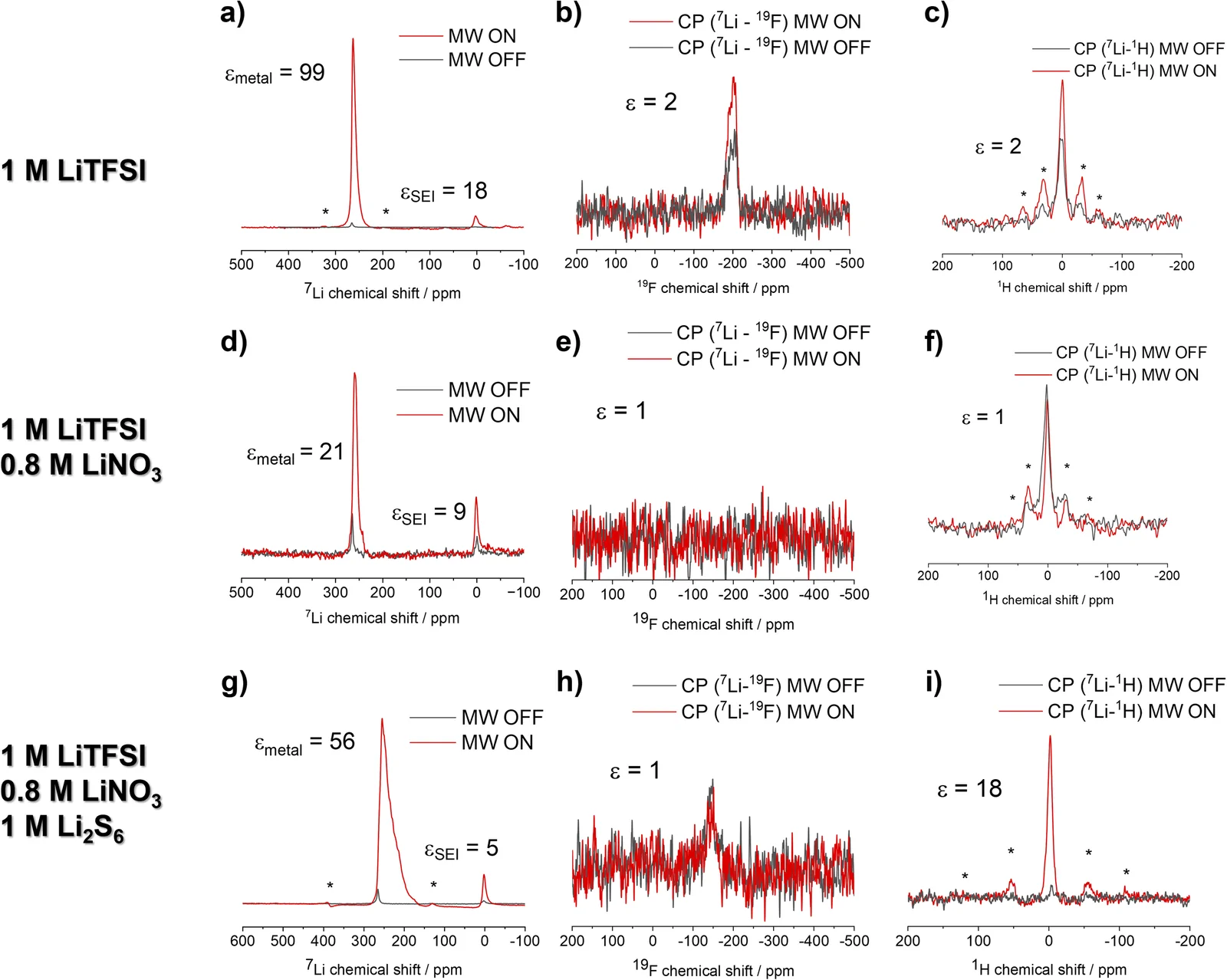
DNP MAS NMR experiments of SEI formed after galvanostatic plating at 0.5 mA cm^−2^ for 100 h in symmetrical Li metal cells. Direct excitation ^7^Li, ^19^F CP (^7^Li) and ^1^H CP (^7^Li) NMR measurements of the SEI with (red) and without (grey) microwave irradiation collected from cells containing different electrolytes: (a–c) 1 M LiTFSI, (d–f) 1 M LiTFSI + 0.8 M LiNO_3_ and (g–i) 1 M LiTFSI + 0.8 M LiNO_3_ + 1 M Li_2_S_6_ in DOL : DME. Spinning sidebands are marked with asterisks.

Selective enhancements of SEI species formed in electrolytes without and with LiNO_3_ (0.8 M) and poly-S are observed in ^7^Li, and double resonance (CP) ^7^Li–^1^H and ^7^Li–^19^F experiments. ^7^Li MAS NMR spectra show a noticeable enhancement of the peak area (*ε*) of the lithium metal signals during MW irradiation (Fig. 4a, d and g). Furthermore, the enhanced Li metal signals are characterized by a broadening towards lower chemical shifts (referred to as the “tail”) resulting from partial saturation of the conduction electron spin resonance (CESR) transition, reducing the Knight shift.^30,58^ The observed hyperpolarization varies significantly (*ε* = 21 to 99) across samples plated in different electrolytes. Maimon *et al.*^59^ have, in dendrites formed in solid-state polymer matrices, shown that dendritic structures generate higher enhancements than bulky microstructures, since these microstructures are more uniformly penetrated by the microwaves.^59^ The values of *ε* seen here are consistent with this assignment and in agreement with the *operando* NMR and SEM results, the electrolytes without LiNO_3_ and with poly-S leading to more microstructure formation than electrolytes with LiNO_3_ (Fig. 3, S7 and S9). The variations in the broadening of the “tail” are a measure of the variation in microwave power “seen” by the Li metal particles across the sample. The sharper more symmetric resonances indicate that Li metal particles experience similar levels of microwave power across the sample. The saturation of the CESR transition is poor in these samples, as the change in the Knight shift is small on application of the microwave irradiation (Fig. 4a and d). By contrast, a broad tail indicates that the Li particles across the sample experience a range of microwave power, with a few of them experiencing relatively high microwave powers, as their Knight shift is reduced by almost 100 ppm, shifting towards the lower chemical shift region (Fig. 4g).

The ^7^Li signals at ∼0 ppm are SEI components and residual electrolyte (Fig. 4a, d and g) that do not show any isotropic hyperfine interactions with the conduction electrons of the metal, but the detected signal none-the-less experiences enhancement (*ε* = 5–18), which can be explained by an indirect polarization transfer mechanism, such as spin diffusion or chemical exchange between Li^0^ and Li^+^ ions, or direct polarization from the conduction electrons.^60^ To gain more information about the SEI composition, polarization transfer experiments (CP) from ^7^Li to ^19^F and ^1^H are conducted. The ^7^Li–^19^F CP spectra (Fig. 4b, e and h) show both different signals and different intensities depending on the electrolyte. In the baseline electrolyte with 1 M LiTFSI a signal at −204 ppm is detectable which can be assigned to LiF.^16^ This signal shows an enhancement of *ε* = 2 upon MW irradiation indicating that LiF is located in or close to the inner layer of the SEI and thus within the range of hyperpolarization transfer. However, the relatively low enhancement compared to the ^7^Li SEI signal (*ε* = 18) suggests that LiF is positioned near the outer limit of the polarization transfer range, meaning it is not in the very first SEI layer in contact with the metal. In comparison, the sample gained from the electrolyte containing LiNO_3_ shows no CP signal in the ^19^F spectrum. This suggests that only a small amount of fluorinated species is present within the SEI, too little to be detected by NMR, and that these species are not located in the inner layer of the SEI accessible by the hyperpolarization transfer. The sample containing poly-S, however, shows a signal at −145 ppm, that is not enhanced during MW irradiation. This chemical shift is consistent with decomposition products of LiTFSI with R^1^–CF_2_–R^2^ moieties.^61,62^ This suggests that no fluorinated species located in the inner layer of the SEI are accessible by the hyperpolarization transfer. Therefore, the decomposition products of LiTFSI are likely located in the outer layer of the SEI.

All the ^1^H NMR spectra show a broad signal, but with different peak centers of mass (Fig. 4c, f and i). The ^1^H spectrum without MW from the SEI with LiTFSI without additives shows a peak at ∼3.6 ppm that can be assigned to SEI components originating from the solvents DOL and DME.^16^ On switching the MW on, the signal is enhanced by *ε* = 2 and the centre of the peak is shifted towards ∼−1 ppm. This means that a component with a lower chemical shift, close to the SEI–metal interface, such as LiOH (∼−1.5 ppm),^63^ is enhanced. The ^1^H spectra obtained from the sample containing LiNO_3_ shows a peak center at 1.2 ppm, which is the characteristic chemical shift of alkane moieties, such as those found in organic polymers.^30^ With MW irradiation, the spectrum does not change, indicating that the detected protonated species are not close to the Li metal surface. In contrast to the sample without LiNO_3,_ no LiOH-like species are present within the area accessible by the hyperpolarization transfer. When adding poly-S, the spectrum shows a peak with a chemical shift of ∼−1.9 ppm that can again be assigned to LiOH.^63^ This peak shows no shift, but a significant enhancement of *ε* = 18 when irradiating with MW. Therefore, this detected LiOH is likely to be found close to the surface of the Li metal in the inner SEI.

### Electrochemical performance in Li–S batteries

**Fig. 5:**
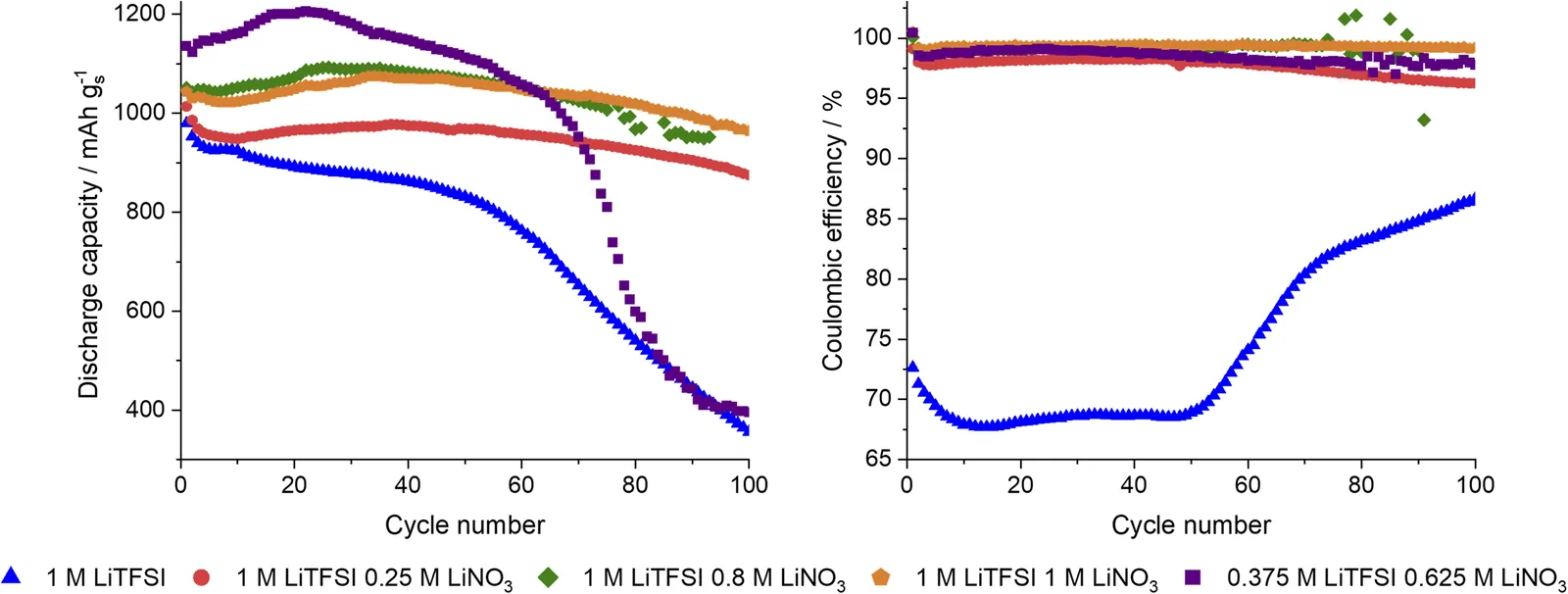
(a) Discharge capacities and (b) coulombic efficiencies of the first 120 cycles of Li–S cells (after a conditioning cycle) with a C-rate of C/10 with the different electrolytes: 1 M LiTFSI (blue), 1 M LiTFSI + 0.25 M LiNO_3_ (red), 1 M LiTFSI + 0.8 M LiNO_3_ (green), 1 M LiTFSI + 1 M LiNO_3_ (orange) and 0.375 M LiTFSI + 0.625 M LiNO_3_ (purple) in DOL : DME.

Major improvements in electrochemical performance are obtained in Li–S batteries containing the LiNO_3_ additive, whereas very poor efficiency and cycle life are obtained without the LiNO_3_ additive (Fig. 5). These trends are fully consistent with the beneficial effect of LiNO_3_ in promoting smooth Li deposition as shown *via* the reduced microstructure formation seen in the *operando*^7^Li NMR (Fig. 2, 3 and S7). Indeed, SEM investigations confirm the beneficial effect of LiNO_3_ on promoting a smoother morphology in Li electrodes obtained from Li–S cells, as well as Li–Ni cells (Fig. S8 and S9). Previous studies have suggested that LiNO_3_ also shows a catalytic effect for the conversion of soluble poly-S to insoluble species at the end of the charge process,^12,13,64^ which would also contribute to the improved performance in the presence of LiNO_3_ observed here.

More detailed inspection of Fig. 5 shows that for Li–S cells with 1 M LiTFSI electrolytes with various concentrations of LiNO_3_ (0.25, 0.8 and 1 M), a capacity close to 1000–1100 mAh g^−1^ is obtained, with a cycle life of around 80–100 cycles for 0.25 and 0.8 M LiNO_3_ and around 100–110 cycles for 1 M LiNO_3_. This suggests that the cause of cell death, in these experiments, is not the consumption of LiNO_3_ as an SEI forming agent, since, if that was the case, increasing the LiNO_3_ would have a more dramatic effect on the cycle life. Chu *et al.*^65^ reported that increasing the LiNO_3_ content enhances electrolyte's solubility for poly-S. The solvation structure of Li^+^ ions is significantly modified in the presence of high NO_3_^−^ concentrations in the electrolyte, benefiting from the high donor number value of NO_3_^−^. This should facilitate the dissolution of poly-S into the electrolyte and could potentially also account for the observed capacity improvement.^65^

The Li–S cells with the 0.375 M LiTFSI + 0.625 M LiNO_3_ electrolyte give somewhat higher capacities (*ca.* 1200 mAh g^−1^), but a shorter cycle life of around 50–80 cycles. The higher capacity in the initial cycles, with respect to the cells containing 1 M LiTFSI with various concentrations of LiNO_3_, can be explained by the changes in SEI (Fig. S3) and the lower viscosity of the electrolyte (Fig. S6), thus overcoming the mass transport limitations that have been proposed to be the main limiting factor of Li–S battery capacities.^66,67^ The narrower cycle life with this electrolyte can be tentatively ascribed to the higher sulfur utilization (due to the higher capacity), which could produce more severe structural changes in the sulfur electrode, as stated previously.^41^ Further, this electrolyte shows a high amount of Li microstructure formation (Fig. 3), which is likely to be an important contribution to the accelerated degradation in the capacity.

## Discussion

**Fig. 6:**
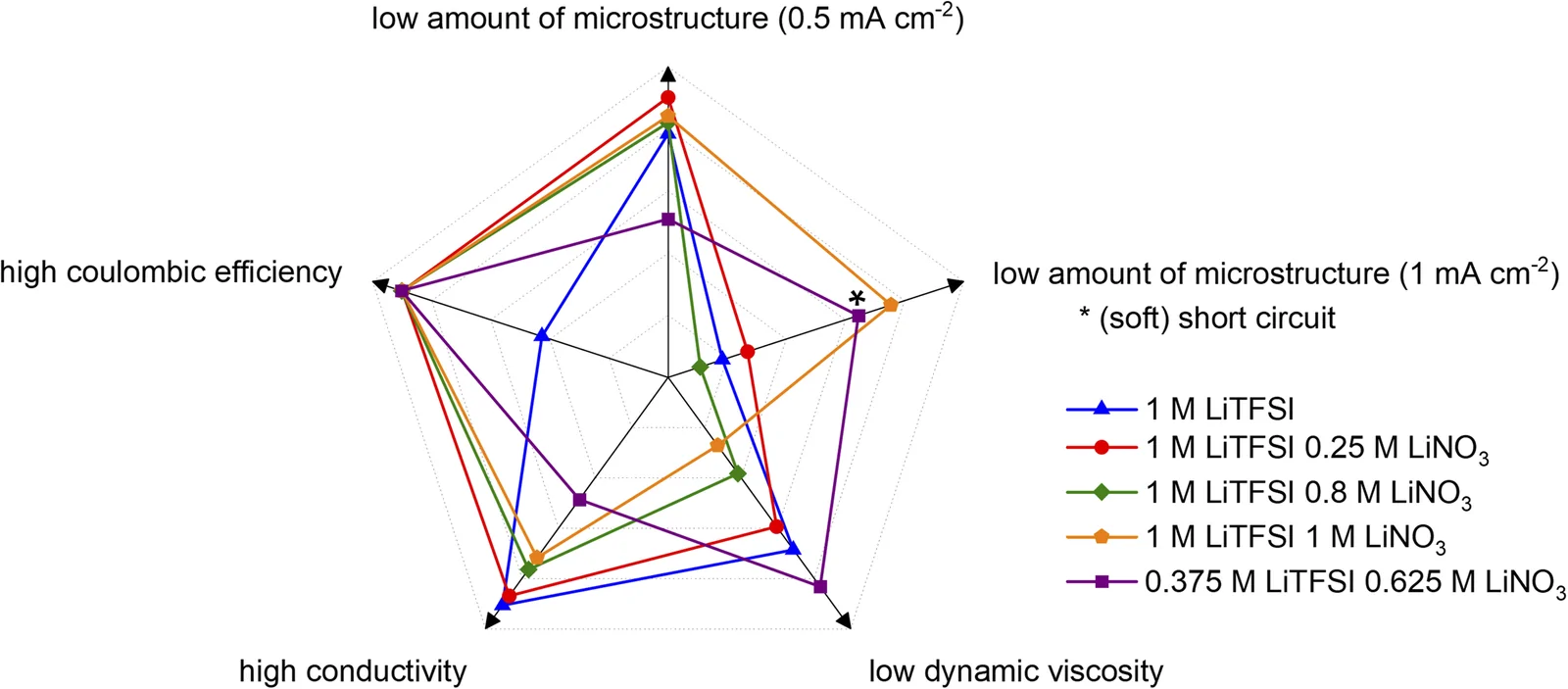
Correlation between the conductivity, dynamic viscosity and the electrochemical behavior (coulombic efficiency of the first cycle) and the amount of plated microstructure in symmetrical Li–Li cells at 0.5 and 1 mA cm^−2^. Comparison of the different electrolytes: 1 M LiTFSI (blue), 1 M LiTFSI + 0.25 M LiNO_3_ (red), 1 M LiTFSI + 0.8 M LiNO_3_ (green), 1 M LiTFSI + 1 M LiNO_3_ (orange) and 0.375 M LiTFSI + 0.625 M LiNO_3_ (purple) in DOL : DME.

It is evident that the electrolyte additive LiNO_3_ strongly affects Li metal deposition. NMR spectroscopy together with SEM imaging shows that, in the absence of LiNO_3_, thinner and more dendritic microstructures form, whereas the presence of LiNO_3_ leads to denser, more compact deposits. This effect is visible on Li metal anodes, Li plated on Ni current collectors and Li electrodes from Li–S cells (Fig. 2, S8 and S9). However, the effect of the electrolyte formulation, and specifically, the LiNO_3_ concentration, is highly complex. This work contributes towards the mechanistic understanding of LiNO_3_ reactions on Li electrodes and helps untangle the complex correlations between the electrochemical performance, Li microstructure, as affected by the SEI, and transport-related bulk electrolyte properties (Fig. 6).

The baseline electrolyte with 1 M LiTFSI exhibits a similar conductivity and viscosity in comparison to the LiNO_3_ containing electrolytes, but a poor electrochemical performance, with very low coulombic efficiency in Li plating/stripping experiments and a high amount of Li microstructure formation during the *operando* NMR experiments (Fig. 6). This indicates that the SEI properties are a major contribution to the lower plating efficiency. In Fig. S3 it is clearly visible that a higher LiNO_3_ concentration increases the resistance of the native SEI over time indicating that LiNO_3_ contributes to the SEI build-up. In addition, stabilization of the growth of the native SEI occurs much slower with LiNO_3_ suggesting a continuous reorganization of the native SEI, that results in a thicker SEI, as previously reported by Li *et al.*^15^ This LiNO_3_-promoted SEI can be correlated with the better electrochemical performance, because of a better surface covering or/and by differences in chemical composition resulting in better ionic conductivity and flexibility. To explain the differences in the Li metal anode performance, additional investigations of the SEI are necessary. DNP MAS NMR measurements of the SEI formed on Li metal show clear chemical differences depending on the electrolyte. This can be explained, as SEI components are mainly decomposition products of the solvation shell of Li^+^ ions.^64,68^

Without LiNO_3_ in the electrolyte the SEI is characterized by LiTFSI, DOL and DME decomposition products, the ^1^H NMR spectra showing organic DOL and DME residues and LiOH. LiOH is a surface species typically formed in the initial passivation layer by a reaction with atmospheric species or formed in the reaction with water residues in the electrolyte.^14^ In addition, LiOH accumulation in the SEI due to a reaction of OH^−^ species resulting from decomposition of solvent molecules cannot be excluded.^69^ In addition, the ^19^F NMR spectrum shows LiF in the SEI, which results from the decomposition of LiTFSI.^70,71^ The enhancement of the ^19^F and ^1^H DNP NMR spectra (Fig. 4b and c) on applying MW irradiation shows that LiOH and LiF are found in the inner SEI.

The mere presence of inorganic SEI components does not necessarily result in high CE and long cycle life (Fig. 1 and 5). Previous studies have demonstrated that optimal cycling performance is achieved when LiF-rich SEI layers are combined with organic components, because organic polymers can function as binders that mitigate LiF-loss and provide the mechanical flexibility required to accommodate volume change during plating and stripping, as mechanical stability of the SEI remains essential for achieving uniform metal deposition.^72,73^ Notably, in the LiF-rich SEI layer derived from the 1 M LiTFSI electrolyte, organic polymeric species capable of fulfilling this binding role were not detected as main SEI components (Fig. 4c). Since LiF is brittle, an SEI dominated by LiF is more susceptible to cracking during volume change providing one explanation for the poor CE.

The SEI composition changes on the addition of LiNO_3_ to the electrolyte, because NO_3_^−^ has a high electron donating ability and so it can squeeze DME and DOL molecules out of the solvation shell of the Li^+^ ions.^64^ Hence, NO_3_^−^ is dragged to the Li metal surface during plating. The strong oxidative nature of NO_3_^−^ leads to spontaneous reaction with Li metal forming Li_2_O, Li_3_N and likely oxynitrides.^64^ The absence of any ^7^Li shifts >2 ppm of the SEI signal suggests that the species are likely oxynitrides and less Li_3_N. This is in accordance with the detection of more polymeric compounds from solvent reactions and less LiOH (^1^H NMR) and LiF (^19^F NMR) in the SEI (Fig. 4e and f). As no enhancement is detectable in the ^1^H and ^19^F NMR spectra, we can assume that the inner SEI is characterized by inorganic ions that are not detectable with these nuclei. However, the combination of inorganic O- and N-containing SEI components with the detected polymeric organic species (Fig. 4f) provides both good flexibility and sufficient ionic conductivity of the SEI. This combination helps explain the high CE observed for electrolytes containing LiNO_3_ (Fig. 1).

Varying the LiNO_3_ concentrations produces significant alterations in the Li metal electrode reactions. In Fig. 6 it is clearly visible in the *operando* NMR experiments that the electrolyte with 0.375 M LiTFSI + 0.625 M LiNO_3_ shows the worst performance during unidirectional plating. In the experiment with 0.5 mA cm^−2^ it shows the highest amount of microstructure formation and at 1 mA cm^−2^ the fastest cell degradation with a short circuit after 6C. This electrolyte composition shows lower conductivity in comparison to the other LiNO_3_ containing electrolytes, while the other properties are similar. This lower conductivity can lead to starvation of Li^+^ ions on the surface during the plating leading to dendritic growth. Furthermore, this can influence the SEI healing capability, as the SEI forming components are transported in the solvation shell with the Li^+^ ions.

1 M LiNO_3_ shows the best electrochemical performance in comparison to the other investigated electrolytes, with low microstructure formation during unidirectional plating and high coulombic efficiency in the first cycle. However, it shows the highest viscosity and, therefore, second lowest conductivity. In addition, higher cost, due to high salt concentration for electrolyte manufacturing needs to be considered as a drawback. The 0.25 M LiNO_3_ containing electrolyte shows a balance between good conductivity, low viscosity, low salt concentration (price) and good plating efficiency with a high coulombic efficiency and low amount of microstructure formation. The influence of the poly-S needs to be considered in the ranking of the electrolyte performance, as the Li–S cell performance relies heavily on the plating efficiency and the S-redox activity: with poly-S in the electrolyte the Li^+^ ion solvation shell and, therefore, the SEI is altered again. In the presence of poly-S the Li^+^ ions share coordination between the sulfur atoms of the poly-S chains and the O atoms of the ether solvents and NO_3_^−^. Furthermore, cluster formation is favored in DOL : DME solvents meaning one Li^+^ ion is coordinated to two or more poly-S chains.^74^ Therefore, the poly-S can influence the electrolyte Li metal reaction and may show a synergistic effect in the decomposition of LiTFSI. This decomposition reaction explains the detection of R^1^–CF_2_–R^2^ moieties in the ^19^F NMR, and which goes in hand with the formation of Li_2_S and Li_*x*_SO_*y*_, species that were not detectable with the applied measurements.^6,75^ However, the signals of the detected F-species are not enhanced in the DNP experiment and thus are not a component of the inner SEI. In the ^1^H NMR spectrum, however, a significant enhancement of the LiOH signal is detectable, indicating that it is a component of the inner SEI generated by the previously described reactions. This and the higher viscosity and lower conductivity with poly-S addition,^53^ helps to explain the higher microstructure formation during unidirectional plating at both current densities in comparison to the best performing electrolytes (Fig. 3). Therefore, when full Li–S cells with the different additive concentrations are cycled, it is clear that with addition of LiNO_3_ better capacity retention is observed indicating an additional beneficial interaction with the S-cathode. It was reported before that LiNO_3_ has a beneficial effect on CEI formation and the S-redox reaction by catalyzing the conversion of long-chain poly-S to S_8_ during the charge process.^12,76^ In the Li–S cells, the addition of LiNO_3_ results in a better capacity retention with a higher concentration. This can be partly explained by the consumption of LiNO_3_ as SEI forming agent. However, this is not the only explanation, because the increase of the LiNO_3_ concentration would have had a more pronounced effect on the cycle life of full Li–S cells. Hence, the bulk electrolyte properties – such as high viscosity and low conductivity – govern Li^+^-ion transport and may counteract the beneficial effects of LiNO_3_ on SEI formation and the S-redox.

## Conclusions

LiNO_3_ is widely used as an additive in Li metal cells, particularly in Li–S batteries, due to its positive influence on SEI formation and S-redox chemistry. However, reported concentrations vary significantly across the literature. In this work, we present the first systematic investigation of LiNO_3_ concentration effects on Li-metal plating behavior, SEI formation, and Li–S full cell performance, combining electrochemical analysis with *operando* NMR and *ex situ* DNP NMR spectroscopy. Our results show that LiNO_3_ markedly alters SEI composition on Li metal, both on the native layer and during plating. DNP NMR reveals a reduced LiF and LiOH content over the concentrations found in the LiNO_3_-free electrolytes, which is likely accompanied by increased Li_2_O and Li_3_N formation, leading to differences in SEI stabilization. Li plating/stripping experiments in Li–Ni cells demonstrate improved coulombic efficiency with LiNO_3_, though LiNO_3_ concentration changes have only minor impact, indicating that SEI modification enhances reversibility independently of additive level. *Operando* NMR confirms reduced dendritic growth with LiNO_3_, but the extent of microstructural formation shows a complex variation with concentration. Notably, the electrolyte containing 0.365 M LiTFSI + 0.625 M LiNO_3_ performs poorly under unidirectional plating, likely due to its low Li^+^ conductivity. NMR measurements also show that the presence of poly-S further modifies the Li SEI and promotes non-uniform deposition.

Performance comparisons across LiNO_3_ concentrations in full Li–S cells reveal that a high LiNO_3_ concentration, especially 1 M, leads to improvements in cycle life, which is correlated with the enhanced suppression of Li microstructure growth seen *via operando* NMR measurements. However, a high LiNO_3_ concentration increases viscosity and reduces conductivity, which would bring performance limitations at higher C-rates, and increases the costs for Li–S battery commercialization. Therefore, electrolyte formulations need to be tailored to the specific requirements for battery application, which will be achieved *via* an optimal balance of low viscosity, high conductivity, minimal dendritic growth, robust Li–S performance, and cost efficiency.

## Author contributions

JBF: conceptualization; investigation (*operando* NMR measurements; Li//Li cycling); data curation; formal analysis; visualization; writing – original draft. MS and SF: investigation (coin cell assembly and electrochemical cycling of Li//Ni and Li//S cells). MJ: investigation (DNP measurements). LF: investigation (viscosity and conductivity measurements). NGA and CPG: conceptualization; project administration; supervision. JBF, MS, MJ, NGA and CPG: interpretation of results; writing – review & editing. All authors provided critical feedback, contributed to shaping the research and manuscript, and approved the final version.

## Conflicts of interest

There are no conflicts to declare.

## Supplementary Material

FD-269-D6FD00047A-s001

## Data Availability

Data for this article, including *operando* NMR and SEM data are available at Zenodo at https://doi.org/10.5281/zenodo.19048399. Ref. 26 and 77–82 are cited in the supplementary information (SI). Supplementary information: details of electrolyte preparation and bulk electrolyte characterization, impedance measurements of the native SEI, and repeat galvanostatic cycling data of Li–Ni cells. In addition, *operando*^7^Li NMR measurements of lithium plating in poly-S-containing electrolytes and lithium corrosion experiments are presented to elucidate interfacial processes and lithium metal reactivity. Post-mortem SEM characterization of anodes recovered from Li–Ni and Li–S cells is also provided to correlate electrochemical performance with electrode morphology. See DOI: https://doi.org/10.1039/d6fd00047a.
