# Supplementary material for: NMR spectroscopic investigation of LiNO3-induced SEI modification in Li–S batteries: a concentration-dependent study
Source: Faraday Discuss. 2026 May 18. Online ahead of print. doi: 10.1039/d6fd00047a (PMC13411077; doi:10.1039/d6fd00047a)
Supplement: FD-OLF-D6FD00047A-s001 [file FD-OLF-D6FD00047A-s001.pdf]

Supplementary Information (SI) for Faraday Discussions.
This journal is © The Royal Society of Chemistry 2026

Supporting Information

NMR Spectroscopic Investigation of $LiNO_3$-Induced SEI Modification in Li–S Batteries: A Concentration-Dependent Study

Jana B. Fritzke[a,b,c], Mark Stockham[b,d], Marie Juramy[a,b], Samuel D. S. Fitch[b,d], Liam Furness[b,d], Nuria Garcia-Araez[b,d] and Clare P. Grey[a,b],

a Department of Chemistry, University of Cambridge, Lensfield Road, Cambridge CB2 1EW, United Kingdom

b The Faraday Institution, Harwell Campus, OX11 0RA, Didcot, United Kingdom

c Chemistry of Interfaces, Department of Civil Environmental and Natural Resources Engineering, Luleå University of Technology, SE-97187, Luleå, Sweden

current address: Jana.beatrice.fritzke@associated.ltu.se

d School of Chemistry and Chemical Engineering, University of Southampton, SO17 1BJ, Southampton, United Kingdom

1. ELECTROLYTE PREPARATION

The salt concentrations in the electrolytes are reported as moles of salt per liter of solvent mixture, except for the NMR measurements, in which the concentration refers to moles of salt per liter of solution. The conversion between these two methods of reporting concentrations is reported in Table S1 showing that, for the electrolyte compositions studied here, the differences are small (between 7% and 14%).

Table S1. Conversion of concentration values reported as moles per liter of solvent or solution, for the electrolyte compositions studied in this work. In all cases, the solvent mixture is DOL:DME, which is omitted in the table for the sake of clarity.

Concentration as moles of salt per liter of solvent	Concentration as moles of salt per liter of solution
1 M LiTFSI	0.88 M LiTFSI
1 M LiTFSI + 0.25 M $LiNO_3$	0.87 M LiTFSI + 0.22 M $LiNO_3$
1 M LiTFSI + 0.8 M $LiNO_3$	0.86 M LiTFSI + 0.69 M $LiNO_3$
1 M LiTFSI + 1 M $LiNO_3$	0.85 M LiTFSI + 0.85 M $LiNO_3$
0.375 M LiTFSI + 0.625 M $LiNO_3$	0.35 M LiTFSI + 0.58 M $LiNO_3$

2. ELECTROCHEMICAL MEASUREMENTS

2.1 Impedance measurements of the native SEI

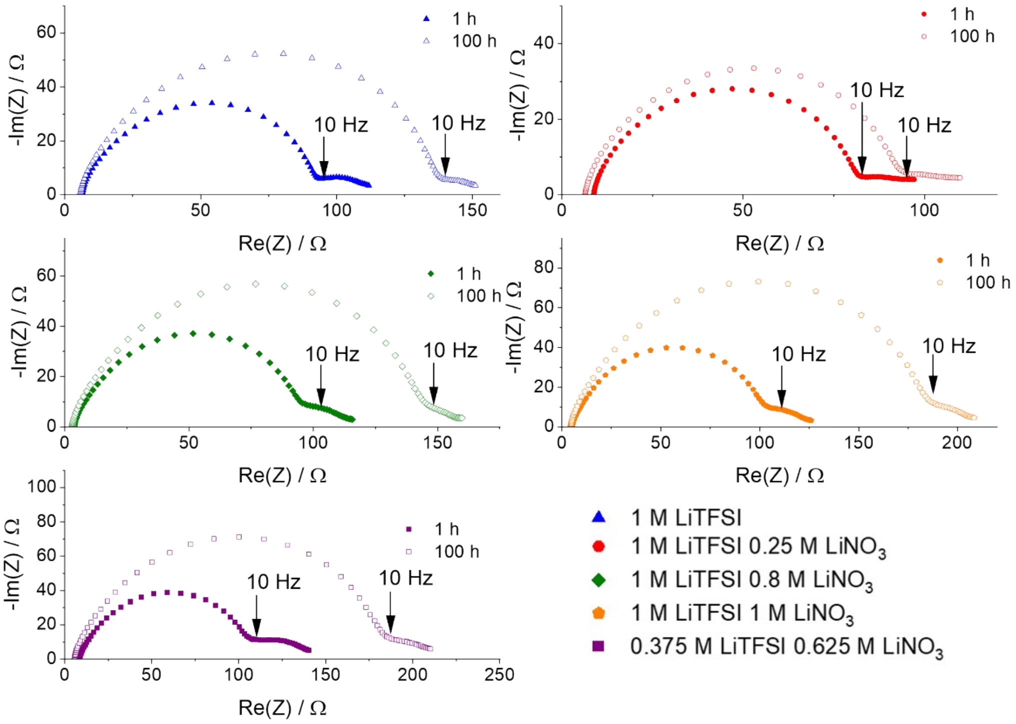

Figure S1. Nyquist plots of impedance measurements of Li-Li symmetrical cells with different electrolytes after 1 h and after 100 h of resting at the open circuit voltage.

To investigate the native SEI on Li metal and the influence of the additive $LiNO_3$, symmetrical Li-Li cells have been measured during rest immediately after assembly by means of potentiostatic electrochemical impedance spectroscopy (PEIS). (Figure S1-S3). The impedance in the different electrolytes reveals a high frequency arc followed by a tail at higher frequencies. The high frequency arc can be ascribed to the ion-transfer resistance through the Li SEI, while the tail at higher frequencies can be ascribed to diffusion processes.[1,2] Fitting the impedance to a simple equivalent circuit model (Figure S2) shows that the capacitance associated with the high-frequency arc is close to ~10 μF, and thus this impedance feature can be ascribed to the compact Li SEI, which under our experimental conditions (using untreated Li electrodes in symmetrical cells) is

originally due to the native passivation layer of the Li electrodes. However, Figure S2 shows that the magnitude of the high-frequency arc increases during the experiments, as the cells are allowed to rest at the open circuit voltage, thus revealing that the ion-transfer resistance of the Li SEI increases over time. Such increase can be explained by reactions of degradation of the electrolyte with the Li electrodes that lead to the deposition of passivating products (e.g. LiF, LiOH, polymeric species, etc.).

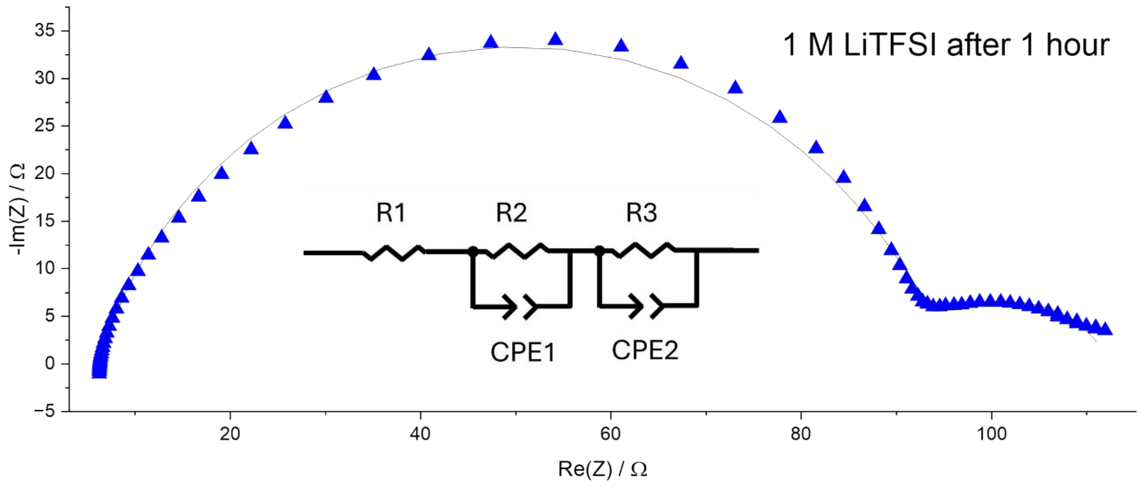

Figure S2. Example of the fitting of the equivalent circuit of the PEIS measurement of a Li-Li symmetrical cells with 1 M LiTFSI in DOL:DME after 1 h of rest at OCV.

Figure S3 plots the magnitude of the impedance at 10 Hz (which is a frequency selected because the high-frequency arc is complete and the contributions from the low frequency tail are minor, see Figure S1) as a function of the resting time. The detected change of the impedance magnitude is tentatively ascribed to the ageing, reorganization and dissolution of the SEI during rest.[3] (Figure S1)

It is clearly visible in Figure S3a that the evolution of the impedance magnitude at 10 Hz depends on the electrolyte composition. The impedance magnitude of the cell with the electrolyte with 1 M

LiTFSI (Figure S3, blue) grows continuously during the first 20 h of the experiment. This could be explained by accumulation of SEI components due to electrolyte decomposition on the Li surface, thus leading to passivating products. After 20 h of rest, the magnitude of the impedance stays constant, indicating that a stable SEI has been formed.

In the cells containing $LiNO_3$, the impedance drops in the first 5-10 h. (Figure S3b) This behavior can be correlated with the dissolution of the initial SEI/passivation layer accompanied with thinning of the interfacial layer resulting in a lower resistance in comparison to the initial resistance. After 10 h the impedance amplitude of all cells containing $LiNO_3$ increases steadily, indicating the formation and thickening of the native SEI.

The electrolyte 1 M LiTFSI 0.25 M $LiNO_3$ leads to the smallest growth of the SEI on Li, in comparison to higher concentration (0.8 M or 1 M) of $LiNO_3$ or the 1 M LiTFSI baseline electrolyte. These results highlight that LiTFSI decomposes on the Li surface, thus leading to passivating products, but such decomposition can be prevented when $LiNO_3$ is added in a moderate amount. However, higher $LiNO_3$:LiTFSI ratios lead to an increase of resistance, attributable to the formation of more passivation products from $LiNO_3$ reduction on Li, and such $LiNO_3$ reaction is also promoted at lower LiTFSI concentration, since the electrolyte 0.375M LiTFSI + 0.625 LiNO3 also experiences a high Li SEI resistance growth. This impedance increase stops after ca. 50 h and stabilizes until the end of the experiment (100 h), so we can assume that the $LiNO_3$ additive is the driving force of the growing and thickening of the native SEI on Li metal.

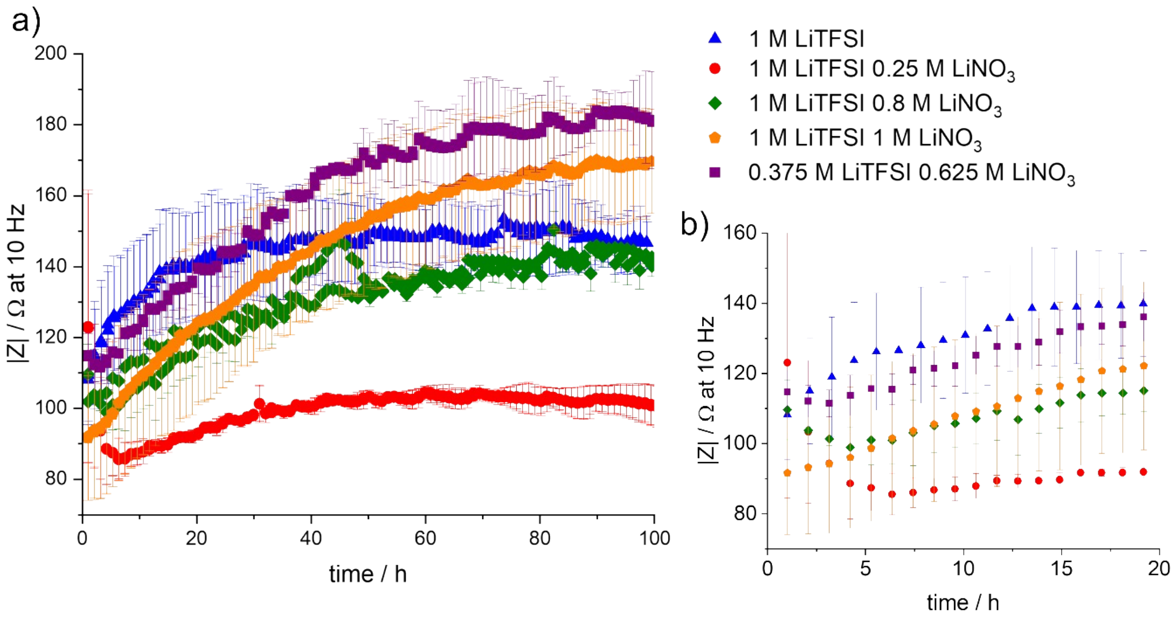

Figure S3. Impedance measurements of the native SEI during OCV. a) Impedance magnitude of the symmetrical Li-Li cell at 10 Hz measured for 100 h in 1 M LiTFSI (blue), 1 M LiTFSI 0.25 M $LiNO_3$ (red), 1 M LiTFSI 0.8 M $LiNO_3$ (green), 1 M LiTFSI 1 M $LiNO_3$ (orange) and 0.375 M LiTFSI 0.625 M $LiNO_3$ (purple) in DOL:DME and b) zoom into the first 20 h of the measurement.

2.2 Galvanostatic cycling in the different electrolyte compositions

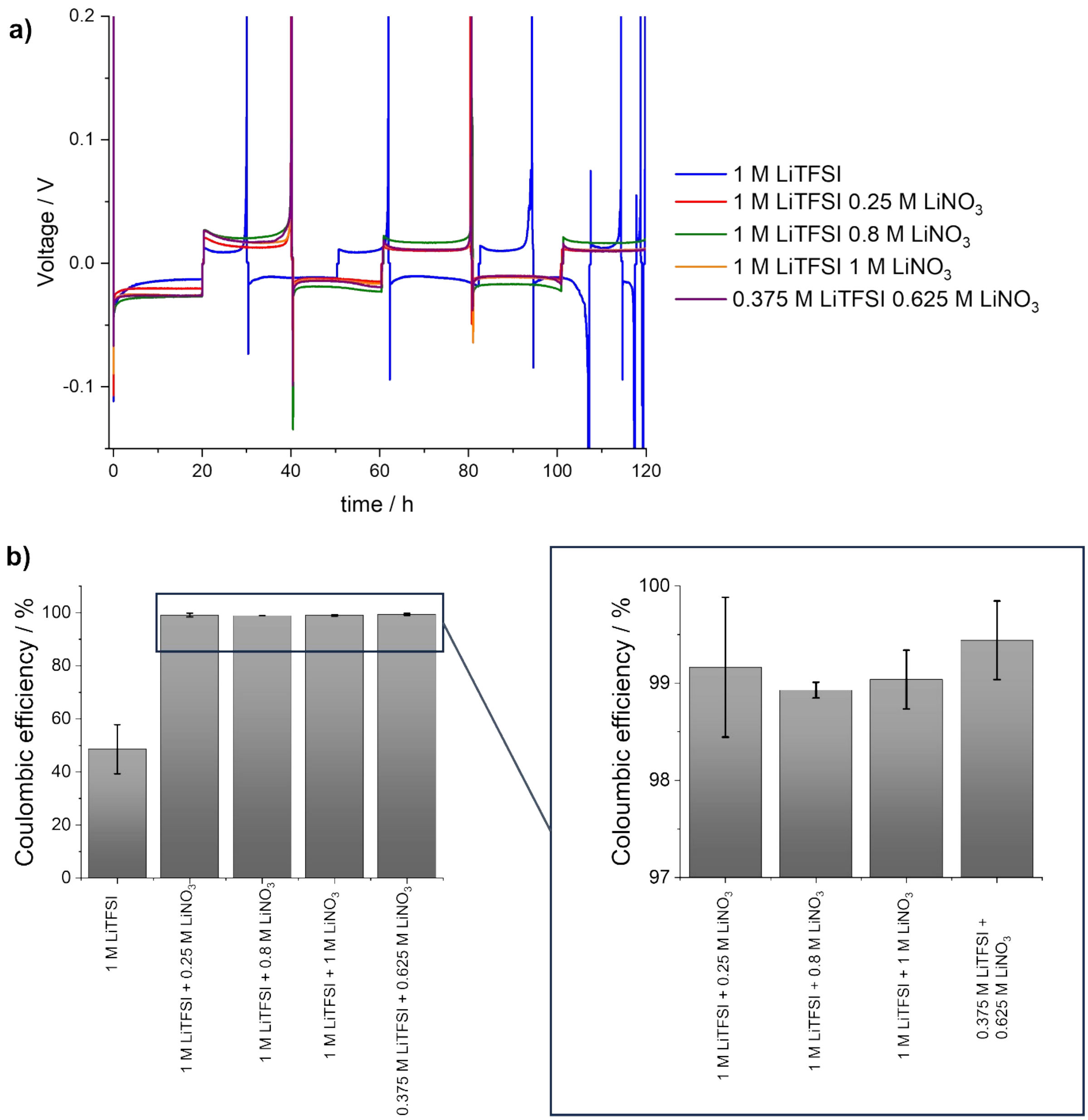

Figure S4. Galvanostatic cycling of Li//Ni cells (repeats) at a current density of 0.5 mA cm^{-2} in different electrolytes. a) Voltage profiles of galvanostatic cycling and b) resulting coulombic efficiencies for the first cycle and a zoom of the best performing electrolytes.

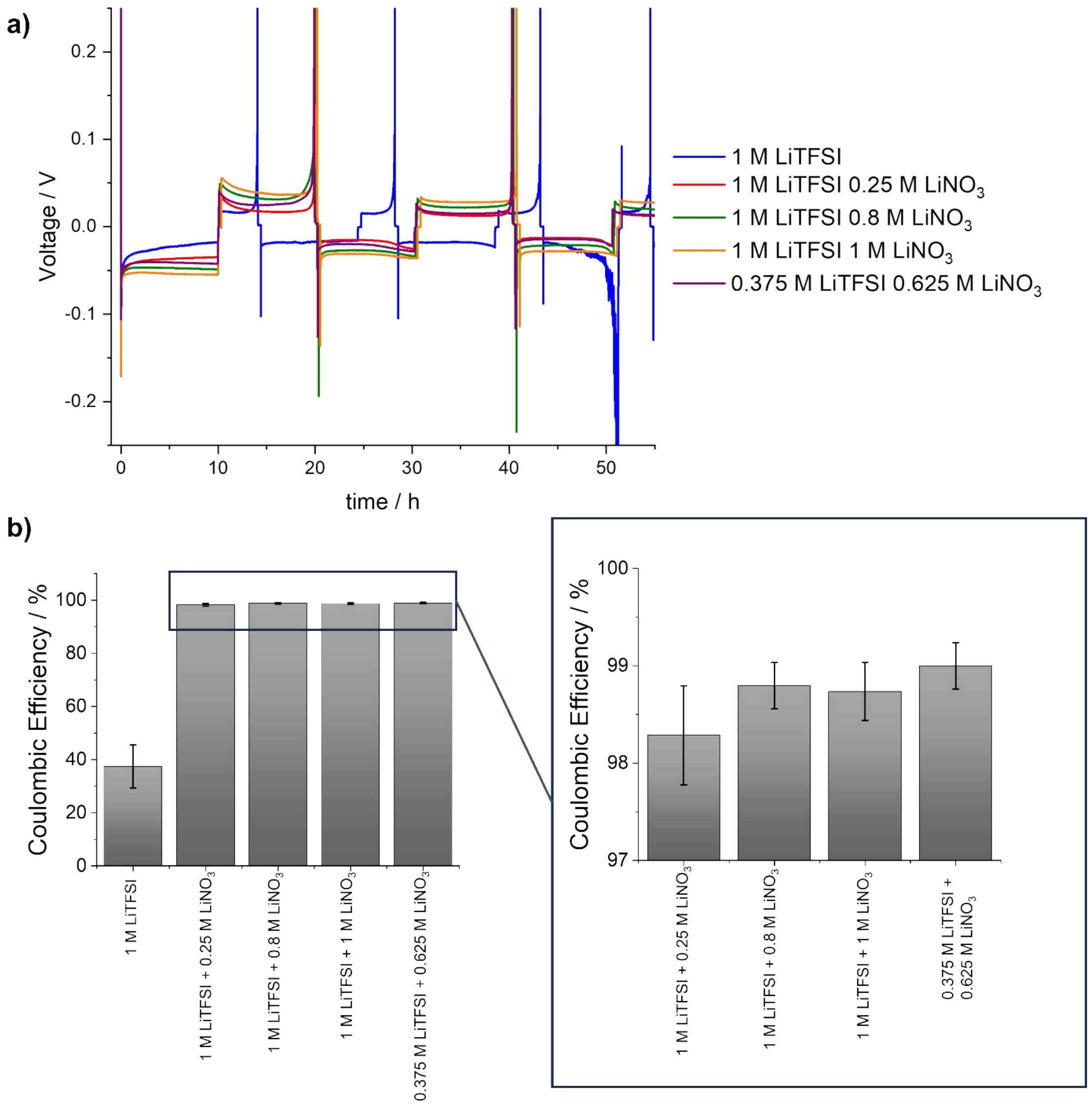

Figure S5. Galvanostatic cycling of Li//Ni cells (repeats) at a current density of 1 mA cm^{-2} in different electrolytes. a) Voltage profiles of galvanostatic cycling and b) resulting coulombic efficiencies for the first cycle and the zoom of the best performing electrolytes.

3. CHARACTERIZATION

3.1 Bulk properties of the different electrolyte compositions

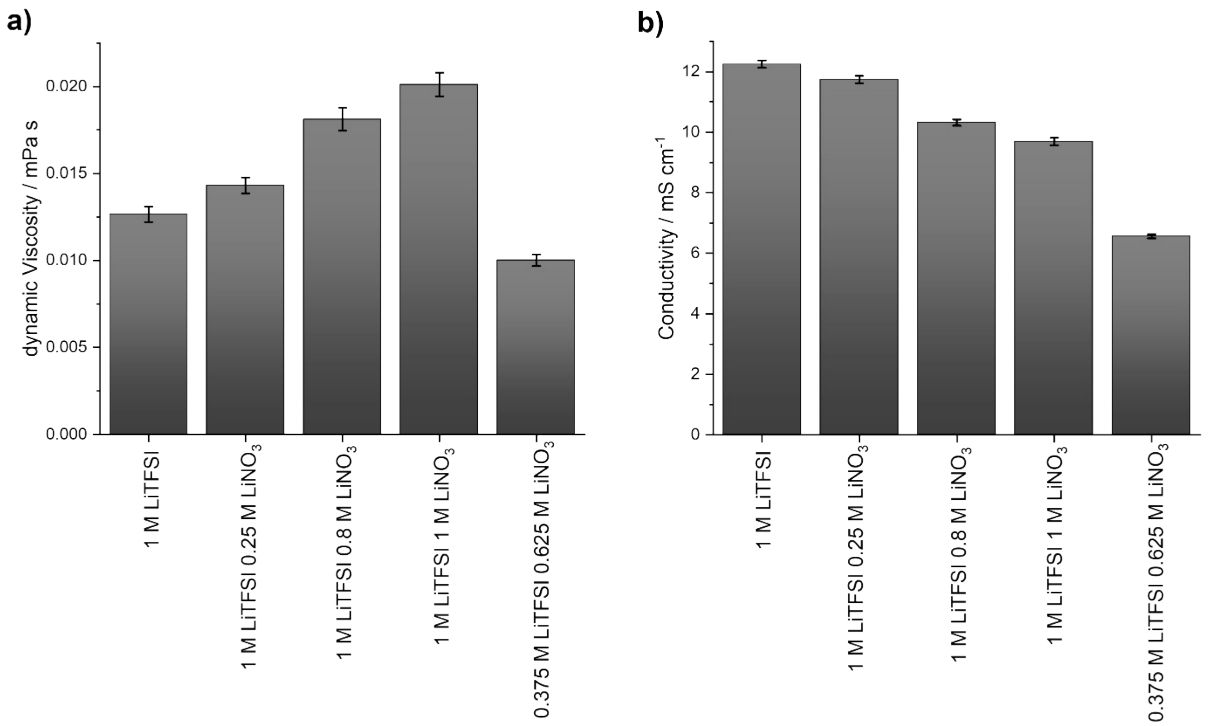

Figure S6. Characterization of the bulk properties of the different electrolytes: a) dynamic viscosity and b) conductivity.

3.2 *Operando* NMR spectroscopy- Li metal plating

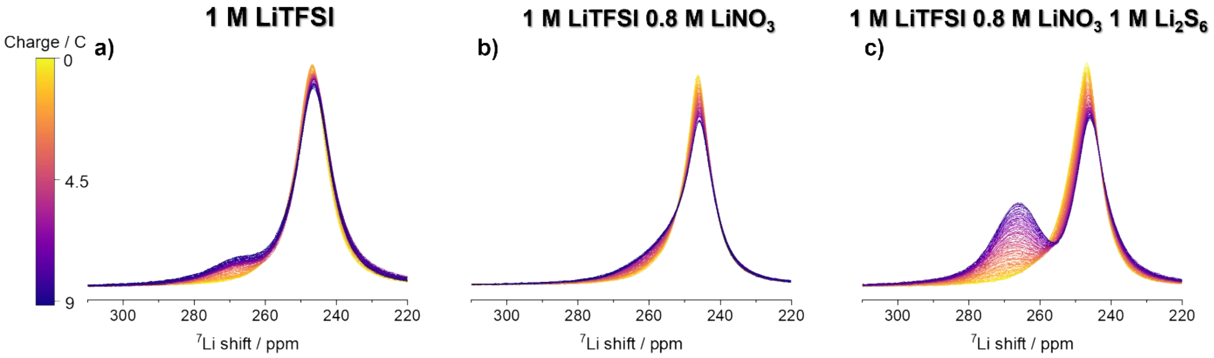

Figure S7. *Operando* NMR experiments of galvanostatic plating at 0.5 mA cm^{-2} in symmetrical Li

metal cells. Change of intensity and shape of the Li metal signal in a) 1 M LiTFSI, b) 1 M LiTFSI 0.8 M $LiNO_3$ and c) 1 M LiTFSI 0.8 M $LiNO_3$ 1 M Li_2S_6 in DOL:DME.

Figure S7 shows that the addition of poly-S to an electrolyte with $LiNO_3$ produces an increased growth of Li microstructures, even higher than in the electrolyte without $LiNO_3$.The combination of $LiNO_3$ and poly-S has been reported to benefit Li metal anode electrochemistry and to prevent Li dendrite formation.[4] However, the high sensitivity of the present *operando* NMR measurements reveal that Li microstructures are promoted by poly-S, likely due to the formation of Li_2S deposits as a result of poly-S reduction, since the insulating nature of Li_2S would produce more inhomogeneity in the local current distribution, thus leading to plating hot spots promoting dendrite/mossy Li formation.

3.3 SEM measurements of plated Li on Ni

Very rough lithium metal deposits are observed when using the 1 M LiTFSI electrolytes without $LiNO_3$. Lithium plating in the electrolyte with $LiNO_3$ produces much flatter deposits, made of bigger grains with more regular grain size than those observed without $LiNO_3$.

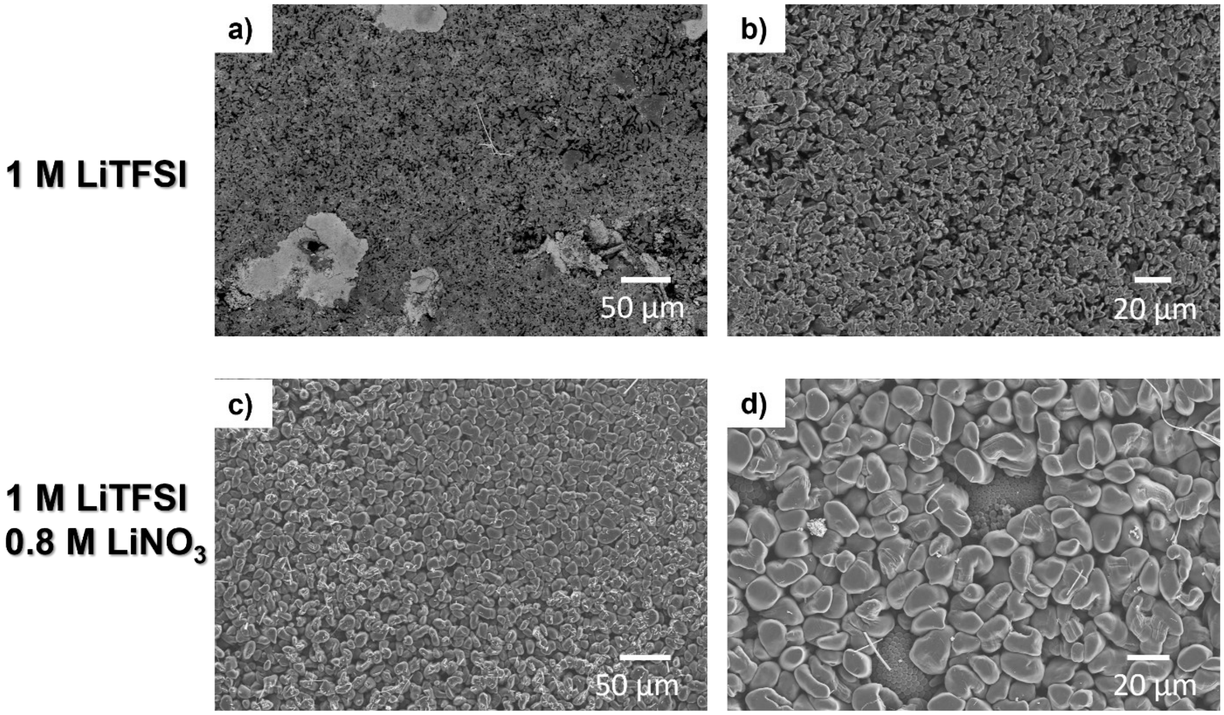

Figure S8. SEM images of the Li metal deposition on Ni current collector at two different magnitudes. The Ni anode was extracted from Li-Ni cells with the different electrolytes 1 M LiTFSI (a and b) and 1 M LiTFSI 0.8 M $LiNO_3$ (c and d) after plating with 1 mA cm^{-2} for 10 h.

3.4 SEM measurements of plated Li in Li-S batteries

In full cells the Li metal deposition shows only minor differences in morphology. The presence of poly-S is influencing the plating performance. The presence of $LiNO_3$ in Li-S cells produces slightly flatter Li anodes with less severe cracks and smaller voids between the particles.

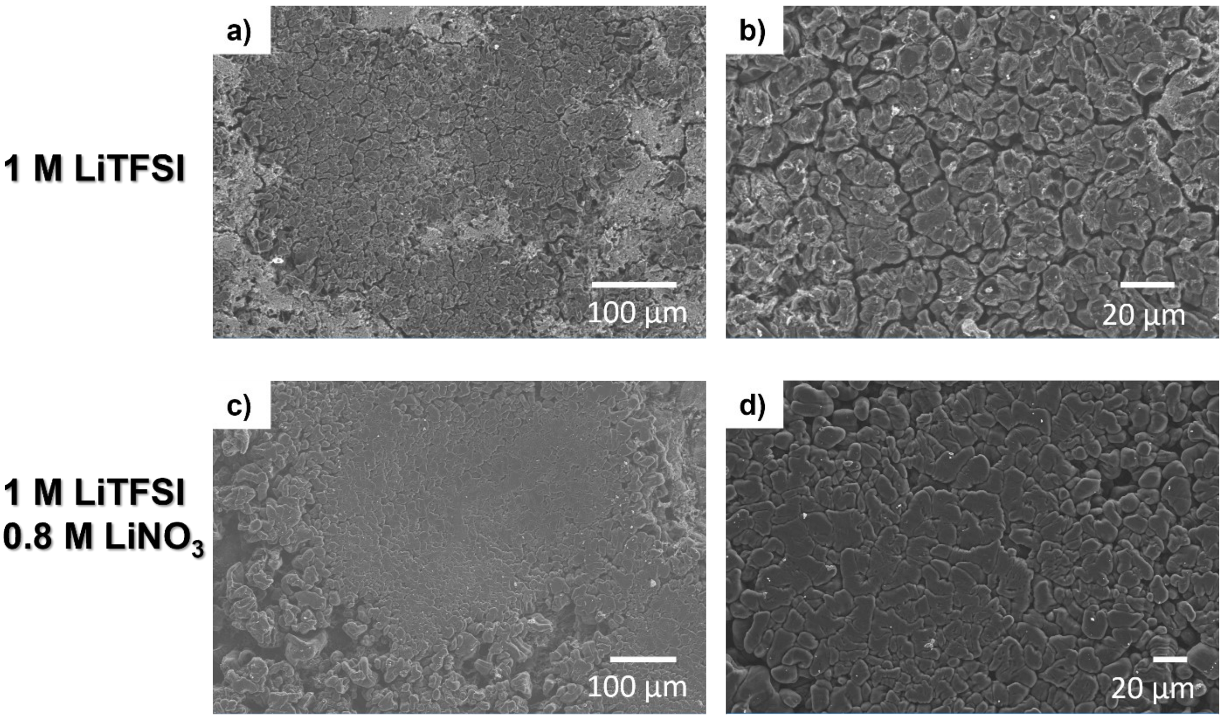

Figure S9. SEM images of the Li metal deposition at two different magnitudes. The Li metal anode was extracted from Li-S cells with the different electrolytes 1 M LiTFSI (a and b) and 1 M LiTFSI 0.8 M $LiNO_3$ (c and d) after 10 cycles at C/10.

3.5 *Operando* NMR Spectroscopy-Corrosion experiments

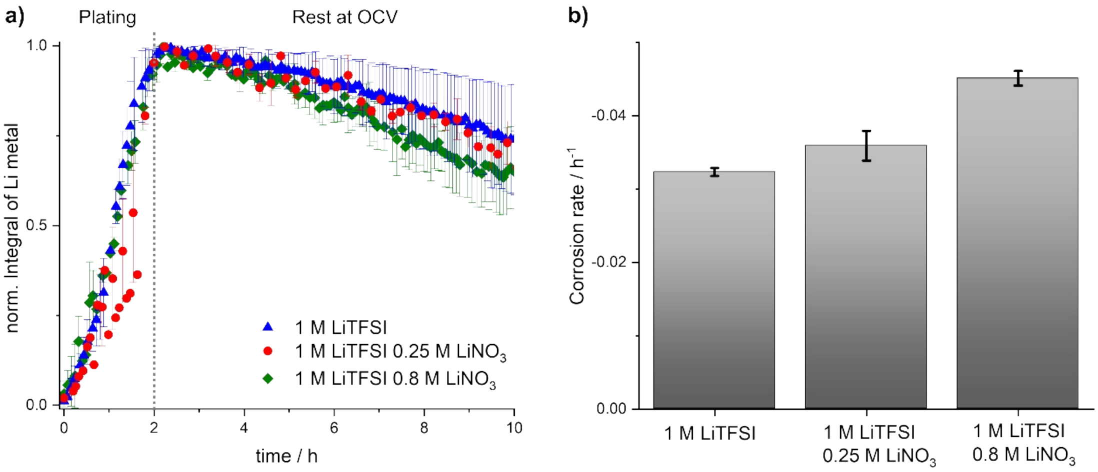

Figure S10. *Operando* ^{7}Li NMR measurement of a Cu//LFP cell in different electrolytes. a)

Evolution of the metal peak integral galvanostatic plating at 1 mA cm^{-2} on Cu in the first 2 h and afterwards resting at OCV. The error bars indicate the distribution of the normalized integral of repeats. b) Calculated corrosion rates from the slope of the integral during OCV.

The decrease in Li-metal signal intensity during the OCV rest period reflects the loss of metallic lithium through corrosion processes. Here, Li corrosion includes both the chemical formation of the SEI on Li metal accompanied by Li oxidation, and the galvanic corrosion that occurs when Li oxidation is coupled to electrolyte reduction on the Cu surface.[5] Mitigating Li dissolution therefore requires not only the formation of a protective SEI on the deposited Li but also effective passivation of the underlying Cu current collector.[6,7] The three investigated electrolytes exhibit nearly identical corrosion rates, indicating that despite differences in chemical composition, none of them provide improved protection against Li corrosion.

REFERENCES

1 S. Drvarič Talian, J. Bobnar, A. R. Sinigoj, I. Humar and M. Gaberšček, *The Journal of Physical Chemistry C*, 2019, **123**, 27997–28007.

2 S. D. Talian, N. Urbanija and M. Gaberšček, *Solid State Ion.*, 2025, **429**, 116987.

3 D. M. C. Ould, S. Menkin, H. E. Smith, V. Riesgo-Gonzalez, E. Jónsson, C. A. O'Keefe, F. Coowar, J. Barker, A. D. Bond, C. P. Grey and D. S. Wright, *Angewandte Chemie International Edition*, 2022, **61**, e202202133.

4 W. Li, H. Yao, K. Yan, G. Zheng, Z. Liang, Y.-M. Chiang and Y. Cui, *Nat. Commun.*, 2015, **6**, 7436.

5 A. B. Gunnarsdóttir, C. V. Amanchukwu, S. Menkin and C. P. Grey, *J. Am. Chem. Soc.*, 2020, **142**, 20814–20827.

6 A. Kolesnikov, M. Kolek, J. F. Dohmann, F. Horsthemke, M. Börner, P. Bieker, M. Winter and M. C. Stan, *Adv. Energy Mater.*, 2020, **10**, 2000017.

7 D. Lin, Y. Liu, Y. Li, Y. Li, A. Pei, J. Xie, W. Huang and Y. Cui, *Nat. Chem.*, 2019, **11**, 382–389.
